# THC and CBD differentially modulated gut microbiota in the Poly I:C rat model of schizophrenia

**DOI:** 10.3389/fphar.2026.1858604

**Published:** 2026-08-10

**Authors:** Natalia Miguel-Ramiro, Nicolás Lamanna-Rama, Marta Casquero-Veiga, Diego Romero-Miguel, Javier Fernández, Cristina Santa-Marta, Karina S. MacDowell, Raquel Chávez-Valencia, Juan C. Leza, Felipe Lombó, Manuel Desco, María Luisa Soto-Montenegro

**Affiliations:** 1 Instituto de Investigación Sanitaria Gregorio Marañón, Madrid, Spain; 2 Departamento de Bioingeniería, Universidad Carlos III de Madrid, Madrid, Spain; 3 Grupo de Investigación “Biotechnology in Nutraceuticals and Bioactive Compounds-BIONUC”, Departamento de Biología Funcional, Área de Microbiología, Universidad de Oviedo, Oviedo, Spain; 4 Instituto Universitario de Oncología del Principado de Asturias (IUOPA), Oviedo, Spain; 5 Instituto de Investigación Sanitaria del Principado de Asturias (ISPA), Oviedo, Spain; 6 Departamento de Física Matemática y de Fluidos, Universidad Nacional de Educación a Distancia (UNED), Madrid, Spain; 7 Centro de Investigación Biomédica en Red de Salud Mental (CIBERSAM), Instituto de Salud Carlos III, Madrid, Spain; 8 Department of Pharmacology and Toxicology, School of Medicine, Universidad Complutense (UCM), IIS Imas12, Instituto Universitario de Investigación en Neuroquímica (Neurochemistry Research Institute UCM) (IUIN), Red Española de Investigación en Estrés (Spanish Network for Stress Research) (REIS), Madrid, Spain; 9 Grupo de Investigación de Alto Rendimiento en Fisiopatología y Farmacología del Sistema Digestivo de la Universidad Rey Juan Carlos (NeuGut-URJC), Madrid, Spain

**Keywords:** CBD - cannabidiol, microbiota-gut brain axis, polyI:C, schizophrenia, THC - tetrahydrocannabinol, maternal immune activation, neurodevelopment

## Abstract

**Background:**

Disruptions in the gut-brain-microbiota axis are increasingly linked to psychiatric disorders. While cannabinoids possess immunomodulatory and neuroprotective properties, their impact on the microbial architecture during critical neurodevelopmental periods remains poorly understood. This exploratory study investigated whether adolescent treatment with delta-9-tetrahydrocannabinol (THC), cannabidiol (CBD) or their combination modulates dysbiosis in the maternal immune activation (MIA) rat model of schizophrenia.

**Methods:**

Pregnant Wistar rats received Poly I:C or saline on gestational day 15. From the offspring of treated rats, eight groups were studied based on phenotype (Saline, MIA) and adolescent treatment (vehicle, CBD, THC, THC +CBD), with caecum contents analyzed via 16S rRNA sequencing.

**Results:**

MIA-offspring exhibited reduced bacterial richness, which was further exacerbated by combined THC + CBD treatment. Notably, CBD was associated with beneficial shifts, including reductions in pro-inflammatory Pseudomonadota, Enterobacteriaceae and Pasteurellaceae. Furthermore, both cannabinoids decreased the pro-inflammatory Bilophila wadsworthia in MIA-offspring while promoting anti-inflammatory, short-chain fatty acid-producing taxa such as Oscillibacter, Roseburia and Prevotellaceae.

**Discussion:**

Exploratory correlation analyses taken at PND120 revealed that THC and CBD differentially shape microbiotabrain structure and microbiota-brain oxidative/inflammatory relationships in a phenotype-dependent manner, with THC and CBD exerting distinct and sometimes opposing effects on redox and inflammatory status. Our findings highlight the microbiota-modulating potential of THC and CBD in a preclinic model of schizophrenia, underscoring the importance of the gut-brain axis given the high prevalence of adolescent cannabis use and its implications for psychiatric vulnerability.

## 1 Introduction

Schizophrenia is a chronic debilitating neurodevelopmental disorder characterized by a heterogeneous spectrum of symptoms that includes cognitive impairments, social withdrawal and psychosis. Although the condition arises from a complex interplay of genetic and environmental factors, increasing evidence points to abnormal immune activity during pregnancy as a remarkable contributor to disease vulnerability. Epidemiological studies have consistently reported that maternal infections during critical windows of fetal development significantly increased the risk of developing schizophrenia later in adult offspring (Brown, 2006; Brown and Patterson, 2011; Choudhury and Lennox, 2021).

Maternal immune activation (MIA) models, most commonly employing the viral mimetic polyinosinic:polycytidylic acid (Poly I:C), have further corroborated this environmental trigger in preclinical research. Poly I:C administration during gestation induces a transient, systemic immune response in the pregnant dam, leading to a cascade of neurodevelopmental alterations in offspring. These include behavioral, neurochemical and structural abnormalities that mirror core aspects of the human disorder (Meyer, 2014; Hadar et al., 2015; Estes and McAllister, 2016; Romero-Miguel et al., 2021a; Romero-Miguel et al., 2021b; Edemann-Callesen et al., 2023). Notably, research in this model has frequently focused on male offspring, which exhibit persistent deficits in executive functions sensorimotor gating and social behavior, alongside neuropathological hallmarks such as dopaminergic dysregulation and neuroinflammation (Hadar et al., 2015; Hui et al., 2018; Casquero-Veiga et al., 2023). While sex-dependent differences in MIA models are increasingly acknowledged, the male offspring at postnatal day (PND) 100 provides a well-characterized framework for studying schizophrenia-like pathophysiology.

Recently, attention has turned toward the gut microbiota as a potential mediator of these outcomes. The microbiota-gut-brain axis represents a bidirectional communication system integrating neural, immune and metabolic signaling. Disruptions in this axis are increasingly associated with psychiatric and neurodevelopmental disorders, including schizophrenia (Ishida et al., 2022) and autism (Li Y. et al., 2024), where the degree of dysbiosis often mirrors the severity of psychiatric manifestation (Grau-Del Valle et al., 2023).

Reflecting these clinical observations, both patients with schizophrenia and MIA rodent models exhibit shared signatures of gut dysfunctions, including reduced microbial diversity, taxonomic shifts and increased intestinal permeability (Castro-Nallar et al., 2015; Nguyen et al., 2019; Romero-Miguel et al., 2023). Such alterations may contribute to systemic inflammation, altered brain development and behavioral abnormalities through mechanisms involving microbial metabolites (e.g., short-chain fatty acids), immune modulation and disruption of the intestinal epithelial barrier (Zheng et al., 2016). Oxidative stress has also been proposed as a key pathogenic mechanism involved in both schizophrenia and gut dysbiosis. Increased levels of reactive oxygen species (ROS), impaired antioxidant defenses and redox imbalance have been documented in patients with schizophrenia and animal models, suggesting that oxidative mechanisms may bridge gut and brain dysfunction (Ermakov et al., 2021; Flatow et al., 2013; Madireddy and Madireddy, 2020).

Within this framework, exogenous factors such as phytocannabinoids have become a focus of research due to their diverse biological activities. Δ9-tetrahydrocannabinol (THC), main psychoactive compound in cannabis, has been studied for its effects on immune function and neural signaling, primarily through CB1 receptor, though its therapeutic use is often limited by psychotropic side effects. In contrast, cannabidiol (CBD), a non-psychotropic derivative, has gained considerable attention for its well-documented immunomodulatory, antioxidant and neuroprotective properties, which are independent of cannabinoid receptor activation (Pertwee, 2008; Campos et al., 2016). Recent clinical and preclinical studies have begun to characterize the influence of cannabinoids on shaping the gut microbiota, modulating microbial composition, gut permeability and inflammation factors that may have downstream consequences on central nervous system function (Thu et al., 2025; Wang et al., 2025). Moreover, the distinct effects of THC and CBD on neuroinflammatory and oxidative pathways suggest that these compounds may differentially influence the microbiota-gut-brain axis, particularly in the context of neurodevelopmental insults (Lamanna-Rama et al., 2024; Romero-Miguel et al., 2023).

Clinical studies indicate that THC can exacerbate psychotic symptoms and impair specific cognitive domains in individuals with schizophrenia (Fu et al., 2021). Neuroimaging and neurobiological investigations further suggest that THC disrupts key neural circuits, including reward pathways and the default mode network (Li X. et al., 2022). In contrast, preclinical studies suggest that THC in adolescence may ameliorate specific sensorimotor gating deficits in the MIA model, highlighting the complex effects of cannabinoids (Lamanna-Rama et al., 2024). On the other hand, CBD has demonstrated potential therapeutic benefits in clinical and preclinical studies, with improvements in symptom severity. CBD treatment has been associated with reductions in positive symptoms, improvements in functional connectivity and beneficial modulation of glutamate and GABA levels in early-onset psychosis and chronic schizophrenia populations (Coccurello et al., 2022). Beyond its central nervous system effects, preclinical and clinical data suggest that CBD may also exert anti-inflammatory actions and promote the restoration of gut barrier integrity, which could mediate anxiolytic and neuroprotective outcomes (Tao et al., 2025).

Despite these promising findings, significant gaps remain regarding how cannabinoid exposure during critical developmental windows influences gut microbial architecture in schizophrenia models. This lack of knowledge is especially pertinent given the rising prevalence of cannabis use during adolescence and its intricate relationship with psychiatric vulnerability. By employing the MIA model as a unique platform to explore these neurodevelopmental perturbations, the present study investigates changes in gut microbiota composition and diversity in adult male offspring. Specifically, we examine whether adolescent treatment with THC, CBD or their combination modulates MIA-induced microbial alterations. By characterizing microbial profiles in MIA and control offspring under different exposure conditions, we aim to determine whether these phytocannabinoids attenuate, exacerbate, or otherwise reshape MIA-induced dysbiosis. Therefore, this study addresses an underexplored aspect of schizophrenia research, offering a new perspective on the interactions between cannabinoids, the gut microbiota and neurodevelopmental vulnerability.

## 2 Materials and methods

### 2.1 Animals

A total of 96 male Wistar rats were housed under controlled conditions (24 °C ± 0.5 °C) with a 12-h light/dark cycle and *ad libitum* access to food (LabDiet 5L0S, Sodispan Biotech, Spain) and water. Environmental enrichment, including nesting and hiding materials, was provided. All experimental procedures adhered to the European Communities Council Directive 2010/63/EU and the ARRIVE guidelines (du Sert et al., 2020) and were approved by the Ethics Committee for Animal Experimentation of Hospital General Universitario Gregorio Marañón and Community of Madrid (PROEX 020/18). Sample sizes for each experimental condition were determined following the 3Rs principle, assuming independent groups, a detection threshold of 1 standard deviation (SD) and a statistical power of 80%. This study utilizes the animal cohort previously described by Lamanna-Rama et al. (2024), in which the effects of THC on schizophrenia-like abnormalities in the MIA model and the preventive potential of CBD on behavioral, brain structural and biochemical outcomes were assessed at PND120. Structural and biochemical variables from the original dataset were used here to perform the correlation analyses in this work.

### 2.2 Treatments

#### 2.2.1 Maternal immune activation (MIA) model

On gestational day 15, pregnant dams were intravenously administered either Poly I:C (4 mg/kg, P0913, Sigma-Aldrich, Germany) or saline (Casquero-Veiga et al., 2021). This model has been extensively documented for its association with increased susceptibility to neurodevelopmental and behavioral abnormalities in offspring, mirroring features observed in schizophrenia spectrum disorders (Haddad et al., 2020; Casquero-Veiga et al., 2021; Casquero-Veiga et al., 2023; Hameete et al., 2021; Juckel et al., 2021). At postnatal day (PND) 21, male offspring were weaned and housed in groups of two to four animals per cage.

#### 2.2.2 THC and CBD administration during adolescence

From PND28 to PND38, corresponding to the periadolescent period, animals received daily intraperitoneal injections of THC (10 mg/kg, Cayman, United States) or vehicle (VH) (Moore et al., 2021; Lamanna-Rama et al., 2024). CBD administration (10 mg/kg, Cayman, United States) or vehicle (Osborne et al., 2017) was initiated on PND35 and continued until PND49, intending to ameliorate or reverse THC-induced microbiota dysbiosis in MIA-offspring. The vehicle solutions consisted of a mixture of ethanol, cremophor and saline (1:1:18) (Lamanna-Rama et al., 2023). During the 3-day treatment overlapping period (PND35-38), animals received two daily intraperitoneal injections, one containing THC (or its VH) and the other containing CBD (or its VH). To minimize local tissue irritation, both injections were administered on opposite sides of the body.

#### 2.2.3 Experimental groups

The animals were distributed into eight experimental groups (N = 10/group) corresponding to all possible combinations of three study factors: MIA model (saline, MIA), THC treatment (VH, THC) and CBD treatment (VH, CBD).

### 2.3 Magnetic resonance imaging (MRI)

At adulthood (PND120), animals were scanned using a 7-Tesla MRI scanner, following a previously published protocol (Lamanna-Rama et al., 2024). Briefly, coronal T2-weighted images were acquired with the following parameters: TE = 33 m, TR = 3,732 m, averages = 2, slice thickness = 0.4 mm, matrix size = 256 × 256 pixels, and FOV = 3.5 cm^2^ × 3.5 cm^2^. T2-weighted images were registered to a common space reference using rigid registration and subsequently used for volumetric analysis. Ventricular volume was manually segmented according to a rat brain atlas (Paxinos and Watson, 2008). The MRI-derived ventricular volumes obtained by (Lamanna-Rama et al., 2024) were used for the current correlation analysis.

### 2.4 Oxidative stress and inflammatory/oxidative markers

After MRI, brain samples were obtained after euthanasia and the frontal cortex was collected, frozen on ice and stored (−80 °C) for biochemical determinations (n = 8 per group). Data for the oxido-nitrosative and inflammatory enzyme inducible nitric oxide synthase (iNOS) and the nuclear transcription factor erythroid 2-related factor 2 (Nrf2) pathway antioxidants (heme oxygenase-1, HO1 and (NAD(P)H quinone oxidoreductase 1, NQO1), previously published (Lamanna-Rama et al., 2024) were used for correlation analyses. Protein expression in cytosolic extracts was determined by Western blot and quantified using the Bradford method. Proteins were separated by electrophoresis and blotted onto a membrane with a semi-dry transfer system. Blots were blocked with 5% BSA (Sigma, Spain) and incubated overnight at 4 °C with specific primary antibodies: rabbit anti-iNOS (sc-650, 1:750 BSA 2%; SCBT, United States), rabbit anti-HO1 (ab68477, 1:1,000; Abcam, United Kingdom), goat anti-NQO1 (sc16464, 1:750 BSA 1%, SCBT, United States) and mouse anti-β-actin (A5441, 1:10000; Sigma, Spain). Appropriate HRP-conjugated secondary antibodies were used for protein detection. All blots were performed at least three times in separate assays. Immunoreactive bands were quantified by densitometry using ImageJ software (NIH, United States), with values normalized to the β-actin loading control.

### 2.5 Genomic DNA extraction and 16S ribosomal RNA sequencing for metagenomics

To assess microbiota composition, frozen intestinal caecum samples, stored at −80 °C, were analyzed. Tissue collection was performed during adulthood in rodents (PND100-110). Genomic DNA (gDNA) was isolated from 200 mg of frozen cecal feces using the E. Z.N.A.® DNA Stool Kit (VWR, Madrid, Spain), ensuring a final yield of at least 200 μL of extracted DNA. The quantification of gDNA was conducted with a BioPhotometer® (Eppendorf, Madrid, Spain) and DNA concentrations were adjusted to 6 ng/μL before further processing. These samples were subsequently subjected to polymerase chain reaction (PCR) amplification targeting hypervariable regions of the bacterial 16S rRNA gene (V2-4, V3-6, V7-9). Amplification following the manufacturer’s protocol outlined in the Ion 16S Metagenomics Kit (Thermo Fisher Scientific, Madrid, Spain). Amplification products were uniquely indexed using Ion Xpress Barcode Adapters, enabling differentiation between individual samples.

Template preparation for sequencing was carried out using the ION OneTouch 2 System and the ION PGM Hi-Q OT2 Kit (Thermo Fisher Scientific, Madrid, Spain). The sequencing process was performed on the ION PGM System with the ION PGM Hi-Q Sequencing Kit (Thermo Fisher Scientific, Madrid, Spain). The study sequencing was conducted on ION 318 v2 chip, with multiple barcoded samples processed per chip.

### 2.6 Phylogenetic analysis

Consensus data files for each metagenomic sequencing experiment were obtained from the ION Reporter software (version 5.6, Life Technologies Holdings Pte Ltd., Singapore). These spreadsheets provided taxonomic distribution percentages at various levels, facilitating comparative analyses across samples and experimental groups. The taxonomic classification and diversity assessments were performed using the QIIME-2 software (version 2017.6.0).

### 2.7 Statistical analysis

The assessment of normality and homogeneity of variance was conducted using Shapiro-Wilk and Levene’s tests, respectively. For statistical comparisons, three-way ANOVAs were applied, followed by Tukey’s *post hoc* test when the assumptions of normality and homoscedasticity were met. In cases where these conditions were not satisfied, the non-parametric Kruskal–Wallis test was performed, followed by Dunn’s multiple comparisons test. A significance threshold of *p* < 0.05 was adopted across all analyses. To account for multiple comparisons, the Benjamini–Hochberg False Discovery Rate (FDR) correction was applied to all differential abundance tests at each taxonomic level. FDR-adjusted q-values are reported in all Supplementary Tables alongside the original p-values.

All graphical representations were generated using GraphPad Prism (version 8, GraphPad Software, San Diego, CA, United States) and statistical analyses were performed using IBM SPSS Statistics (version 29.0; IBM Corp., Armonk, NY, United States). Data are expressed as mean ± standard error of the mean (SEM) and statistically significant differences are explicitly indicated in the corresponding figures. Effect size is included in the Supplementary Tables to provide a more complete evaluation of the observed effects.

Additionally, analysis of the overall structure and variability of the microbiota communities across the eight experimental animal groups was performed using unsupervised multivariate analysis (PCA) in R software (v4.4.3) with the *prcomp* function on centered and scaled relative data at the genus taxonomic level. Linear Discriminant Analysis (LDA) was applied to identify the most discriminatory bacterial genera between each pair of experimental groups. Prior to LDA, low-variance genera and those present in fewer than 10% of samples were excluded. LDA was performed in R (v4.4.3) using the *lda* function from the MASS package and the bar plots of the top discriminatory genera, ranked by absolute LDA coefficients, were generated using the *ggplot2* function.

Finally, Spearman’s rank correlation coefficient was used to assess ordinal associations between gut microbiota composition and both ventricular volume and biochemical parameters.

## 3 Results

### 3.1 Microbial diversity alterations

**FIGURE 1:**
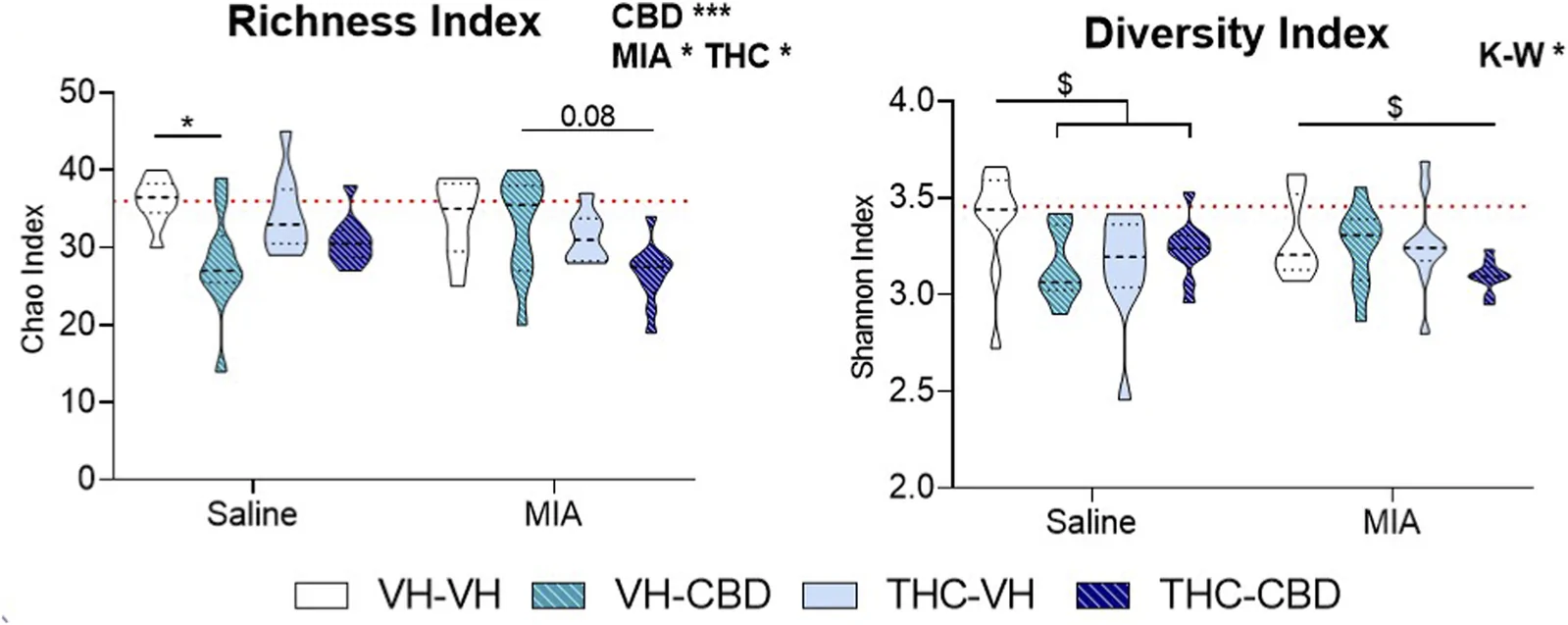
Bacterial diversity richness and diversity. Graphical representation of the changes in bacterial richness and diversity, measured using the Chao and Shannon indexes after THC and CBD treatment (N = 10 per group). Data are shown as mean ± SEM. Three-way ANOVA followed by a *post hoc* test, Tukey’s multiple comparisons (*p < 0.05) or Kruskal–Wallis (KW) analysis followed by Dunn’s *post hoc* test are shown. CBD*** indicates a significant main effect of CBD treatment (p < 0.001) as identified by the three-way ANOVA and MIA × THC* refers to a significant interaction between the MIA model and THC treatment (p < 0.05). Additional statistical significance is indicated by $ *p* < 0.05 by Mann-Whitney U test. Red dotted lines indicate mean value of control group.

ANOVA analysis of bacterial richness as measured by the Chao index (Figure 1) showed a significant effect of CBD treatment (*p < 0.001*), as well as a significant interaction between MIA and THC (*p < 0.05*). Post-hoc comparisons showed a significant reduction in diversity in the Saline-VH-CBD group compared to the control group (*p < 0.05*) and a trend toward reduced diversity in the MIA-THC-CBD group compared to the MIA-VH-CBD group (*p = 0.08*). The Shannon index, which accounts for diversity based on both richness (number of species) and evenness (relative abundance distribution), showed significant differences between groups (Kruskal–Wallis test, *p < 0.05*). Specifically, all the saline offspring treated with cannabinoids exhibited lower richness compared to their control group (*p < 0.05*) and the MIA offspring treated with THC-CBD also showed reduced richness relative to its control group (*p < 0.05*) (Figure 1).

### 3.2 Phylum-level gut microbiota analysis

**FIGURE 2:**
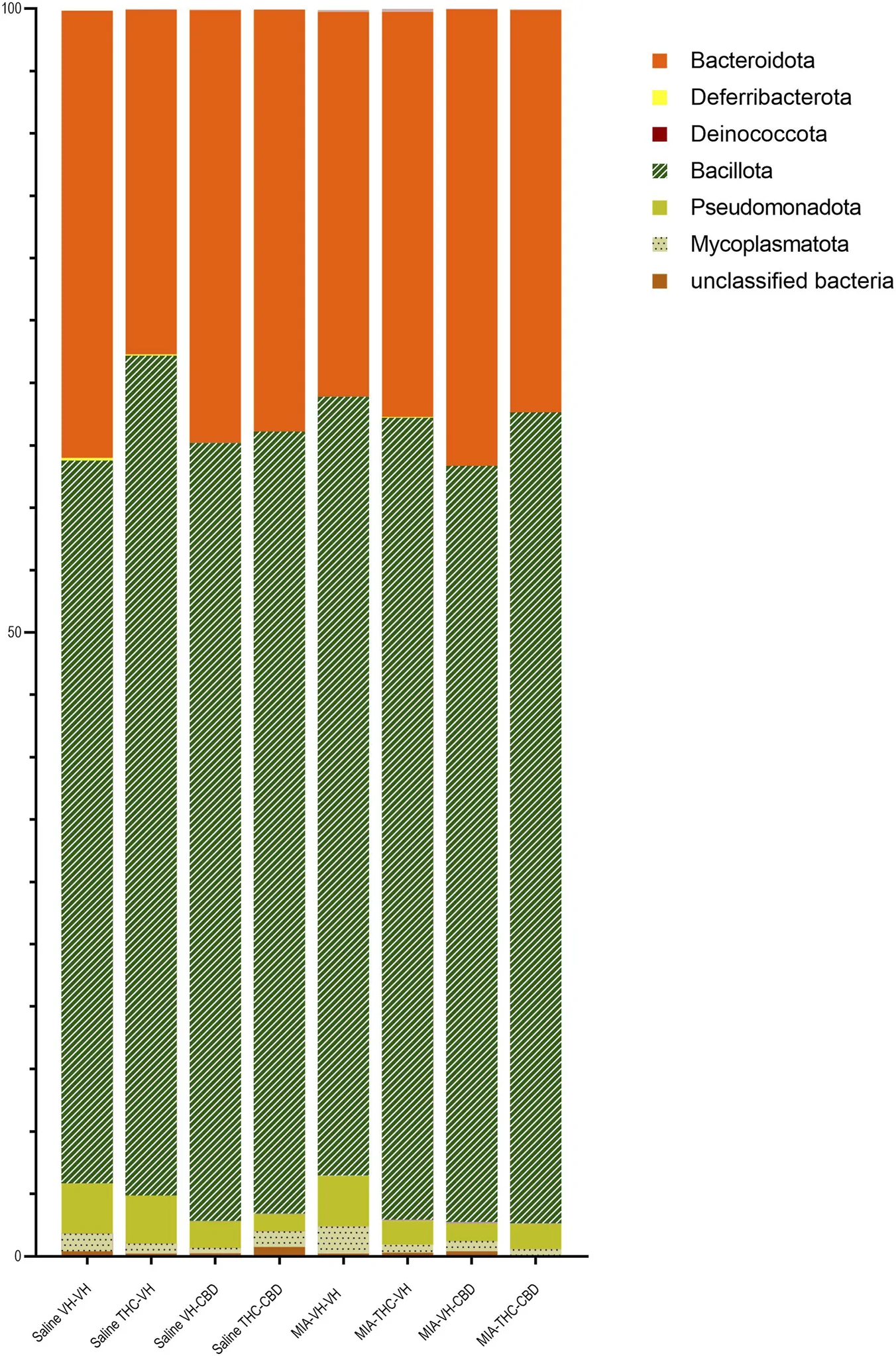
Nested bar plot at the phylum level. Nested bar plots represent the mean relative abundance (%) of the most prevalent bacterial phyla in Saline and MIA groups following treatment with THC, CBD, or their combination (N = 10 per group).

**FIGURE 3:**
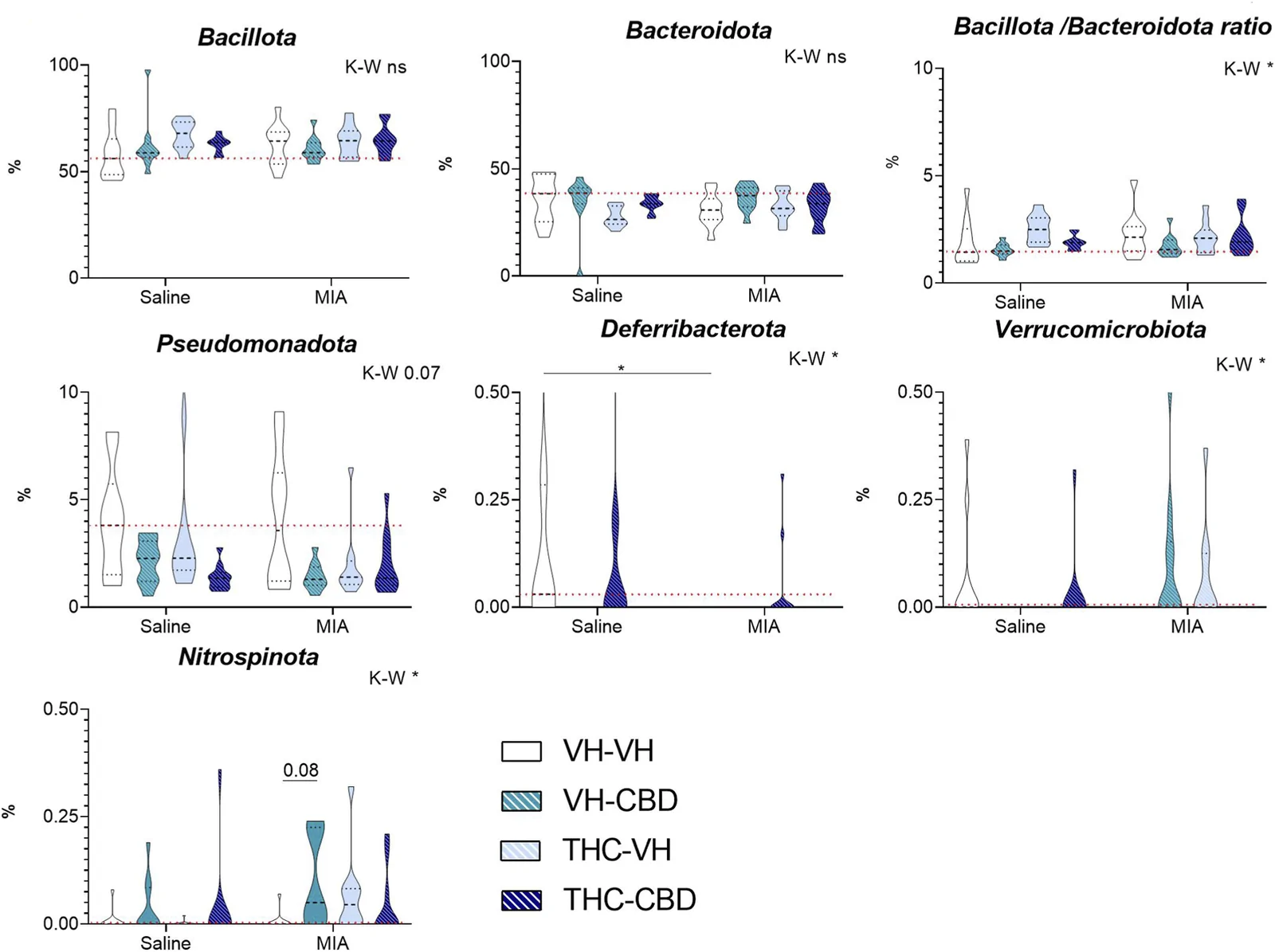
Gut microbiota composition at the phylum level. Graphical representation of the mean percentage of the composition of the most representative phyla and the ratio *Bacillota*/*Bacteroidota* in the Saline and MIA groups following administration of THC, CBD or both (N = 10 per group). Data is shown as mean ± SEM. Kruskal–Wallis test (K–W) followed by Dunn’s multiple comparison are shown (*p < *0.05;* **p < *0.01*; ***p < 0.001). Red dotted lines indicate mean value of control group. Statistical significance was determined after FDR correction.

*Bacillota* and *Bacteroidota* were the predominant phyla across all experimental groups. In the control group (Saline-VH-VH), *Bacillota* represented 63.8% and *Bacteroidota* 35.8%. *Pseudomonadota* was the third most abundant phylum, followed by *Actinomycetota* (Figure 2). In the MIA group (MIA-VH-VH), *Bacillota* increased to 68.9%, while *Bacteroidota* decreased to 30.8% (Figure 2). Given their biological relevance, the *Bacillota*/*Bacteroidota* (B/B) ratio, commonly used as an indicator of gut microbial balance and potential dysbiosis, was assessed. Statistically significant differences in the B/B ratio were found (KW *p < 0.05*) (Figure 3). However, *post hoc* comparisons did not reveal significant differences between MIA-VH-VH and Saline-VH-VH animals (Figure 3).

Statistical analyses found significant group differences in the relative abundance of *Deferribacterota*, *Verrucomicrobiota* and *Nitrospinota* populations. Post hoc analyses showed a significant reduction in *Deferribacterota* in MIA-VH-VH group compared to the control (Saline-VH-VH) (*p < 0.05*). In the presence of CBD, either alone or in combination with THC, *Verrucomicrobiota* was also reduced to undetectable levels. Additionally, *Nitrospinota* population showed a trend toward increased abundance in the MIA-VH-CBD group compared to MIA-VH-VH (*p = 0.08*) (Figure 3). Supplementary Table S1 provides a detailed summary of the statistical analyses in the phyla population.

### 3.3 Family-level microbiota analysis

**FIGURE 4:**
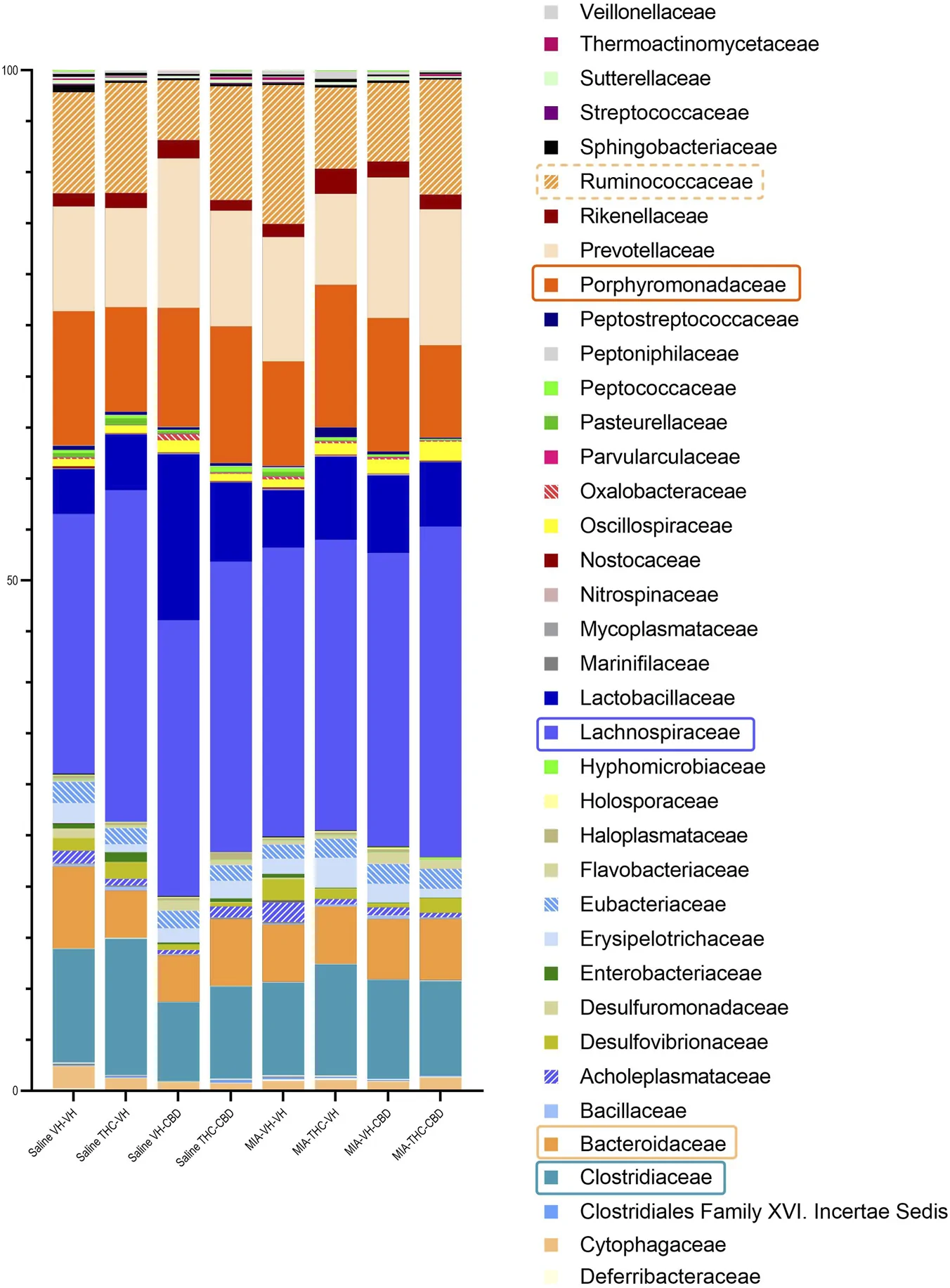
Nested bar plot showing gut microbiota composition at the family level. Graph shows the mean percentage composition of the most representative families for each group (N = 10 per group). Framed families correspond to the most abundant taxa in the gut microbiota. Among these abundant families, those showing statistically significant differences between experimental groups were selected for further in-depth analysis at the genus and species level in subsequent figures.

In the control group (Saline-VH-VH), the most abundant bacterial families were *Lachnospiraceae* (25.42%)*, Clostridiaceae* (13.89%), *Porphyromonadaceae* (13.14%) and *Prevotellaceae* (10.277%). In contrast, in the MIA group, the most prevalent ones were *Lachnospiraceae* (28.28%)*, Bacteroidaceae* (16.25%), *Ruminococcaceae* (13.58%) and *Prevotellaceae* (12.19%). These proportions remained largely stable following THC and CBD treatments, with a few exceptions (Figure 4).

**FIGURE 5:**
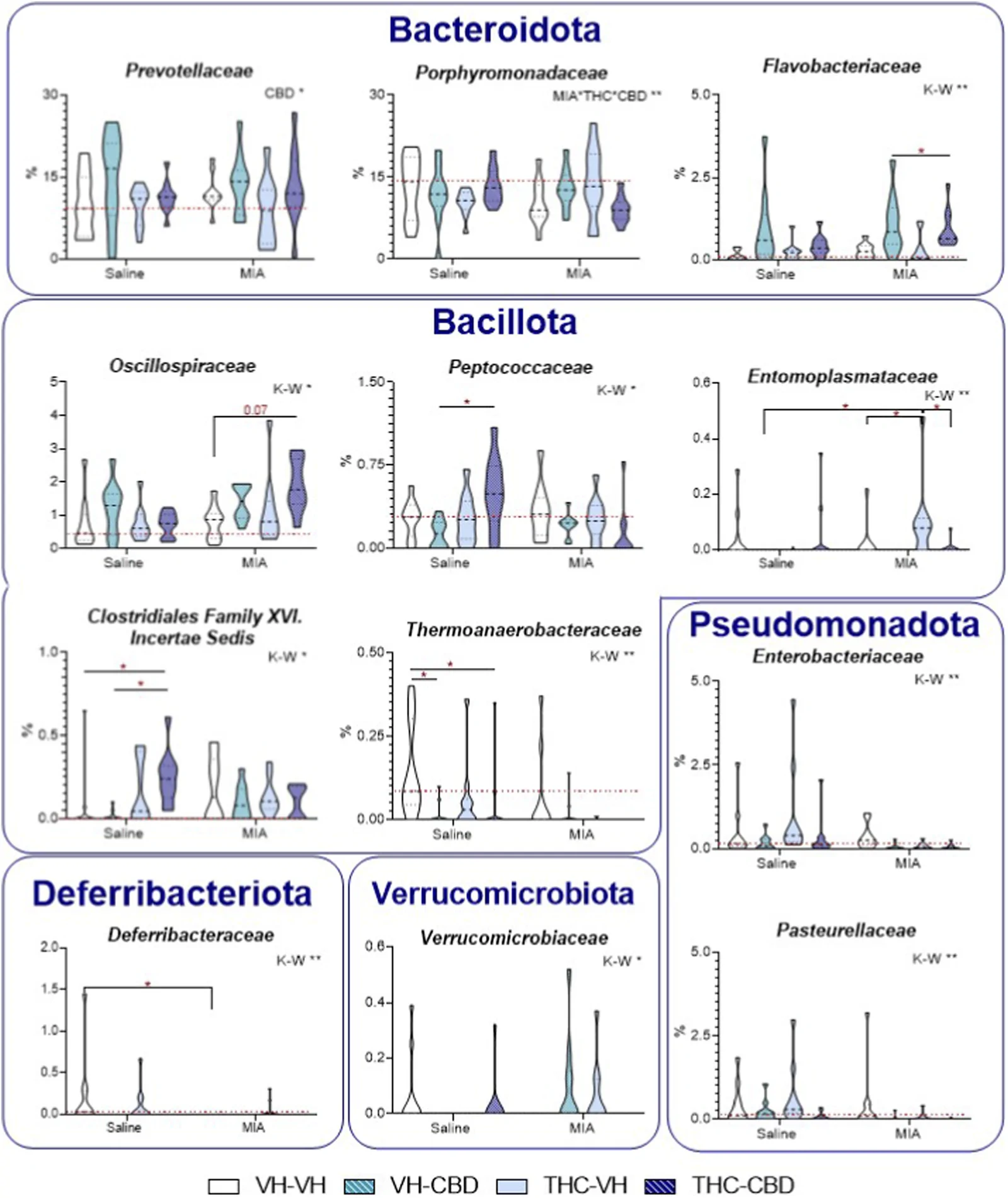
Gut microbiota composition at the family level. The graphs show the average percentage changes in the relative abundance of the most representative bacterial families in the Saline and MIA groups treated with THC, CBD or their combination (N = 10 per group). Data are presented as mean ± SEM. 3-way ANOVA followed by a *post hoc* test, Tukey’s multiple comparisons, or Kruskal–Wallis (KW) analysis followed by Dunn’s *post hoc* test are shown (*p < 0.05, **p < 0.01; ***p < 0.001). Red dotted lines indicate mean value of control group. Statistical significance was determined after FDR correction.

Statistically significant differences between groups were found in several taxa within the phylum *Bacillota in Oscillospiraceae* (p < 0.05), *Peptococcaceae* (p < 0.05), *Entomoplasmataceae* (p < 0.01), *Clostridiales Family XVI* (p < 0.05) *and Thermoanaerobacteraceae* (p < 0.01), with most changes occurring in saline offspring following CBD treatment. Post hoc analysis showed that THC + CBD increased *Clostridiales Family XVI* while decreased *Thermoanaerobacteraceae* in Saline-offspring. Within the phylum *Bacteroidota*, significant group differences were detected in *Prevotellaceae and Flavobacteriaceae families*, both modulated by CBD treatment (*p < 0.05* and *p < 0.01,* respectively). Within *Pseudomonadota, significant group differences were found in Enterobacteriaceae* and *Pasteurellaceae* (both p < 0.01), with reductions in their abundance by both cannabinoid treatments in MIA offspring, while an opposite pattern of increased abundance was observed in Saline-offspring (Figure 5). Detailed population metrics at family level across groups are shown in Supplementary Table S2.

### 3.4 Genus- and species-level microbiota analysis

**FIGURE 6:**
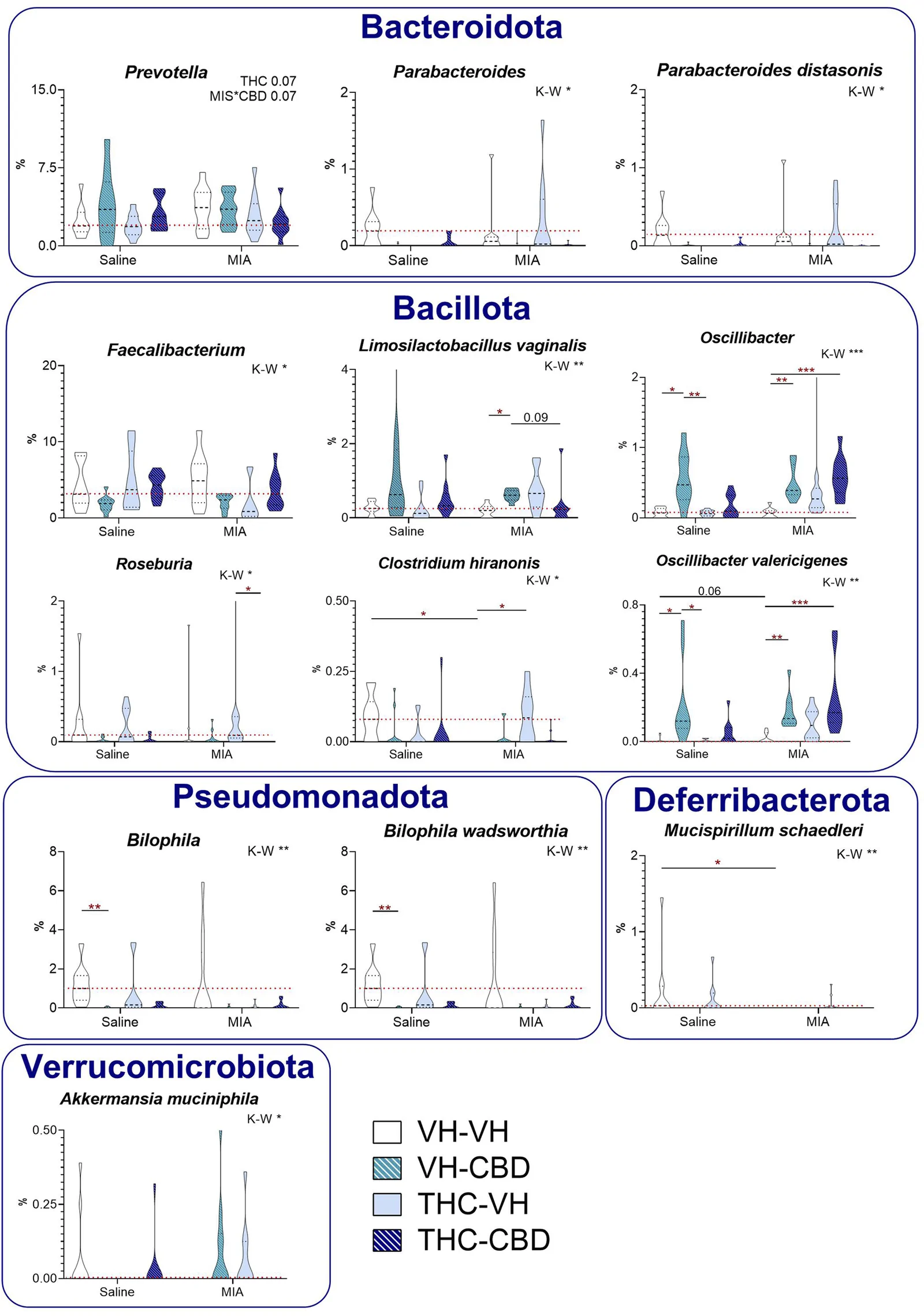
Gut microbiota composition at the genus and species level. Graphical representation of the mean relative abundance (%) of bacterial genera and species within the most representative families in Saline and MIA groups following administration of THC, CBD or their combination (N = 10 per group). Data are presented as mean ± SEM. Three-way ANOVA followed by a *post hoc* test, Tukey’s multiple comparisons, or Kruskal–Wallis (KW) analysis followed by Dunn’s *post hoc* test are shown (*p < 0.05, **p < 0.01; ***p < 0.001). Red dotted lines indicate mean value of control group. Statistical significance was determined after FDR correction.

Within the phylum *Bacillota* (Figure 6), MIA-VH-VH animals exhibited a significant reduction in the abundance of *Clostridium hiranonis (p < 0.05)* compared to Saline-VH-VH group*.* In contrast, treatments with THC and CBD induced notable changes in the relative abundance of specific bacterial genera and species. Significant group differences were identified in *Limosilactobacillus vaginalis* (p < 0.01), *Faecalibacterium* (p < 0.05), *Clostridium hiranonis* (p < 0.05), *Roseburia* (p < 0.05), *Oscillibacter* (p < 0.001) and *Oscillibacter valericigenes* (p < 0.01). Specifically, THC treatment increased the abundance of *Clostridium hiranonis* (p < 0.05) and *Roseburia* (p < 0.01) in MIA-offspring. CBD treatment increased *Limosilactobacillus vaginalis* (p < 0.05) and *Oscillibacter valericigenes* (p < 0.01) in MIA-offspring. The combination of THC and CBD led to increased levels of *Oscillibacter valericigenes* (p < 0.001) in MIA-offspring.

Within the phylum *Bacteroidota* (Figure 6), statistically significant differences between groups were found in *Parabacteroides* (p < 0.05) and *Parabacteroides distasonis* (p < 0.05). THC treatment increased its abundance in the MIA-offspring, whereas CBD treatment, both alone and in combination with THC, reduced their abundance.

Related to the phylum *Pseudomonadota* (Figure 6), significant differences between groups were found in *Bilophila* (p < 0.01) and *Bilophila wadsworthia* (p < 0.01), with both cannabinoids reducing their abundance in both models. Within the phylum *Deferribacterota* (Figure 6)*,* significant differences between groups were found in *Mucispirillum schaedleri* (*p < 0.01*). Notably, both taxa were undetectable in untreated MIA-offspring compared to untreated Saline-offspring (*p < 0.05*). However, THC treatment increased its abundance in both phenotypes (Figure 6).

Lastly, in the phylum *Verrucomicrobiota*, significant group differences were found in *Akkermansia muciniphila* (p < 0.05). This species was undetectable in untreated MIA-offspring and showed a numerical increase following THC or CBD treatment. Detailed population metrics at genus and species level across groups are shown in Supplementary Table S3.

### 3.5 PCA of the Bray-Curtis distance of the microbiota

**FIGURE 7:**
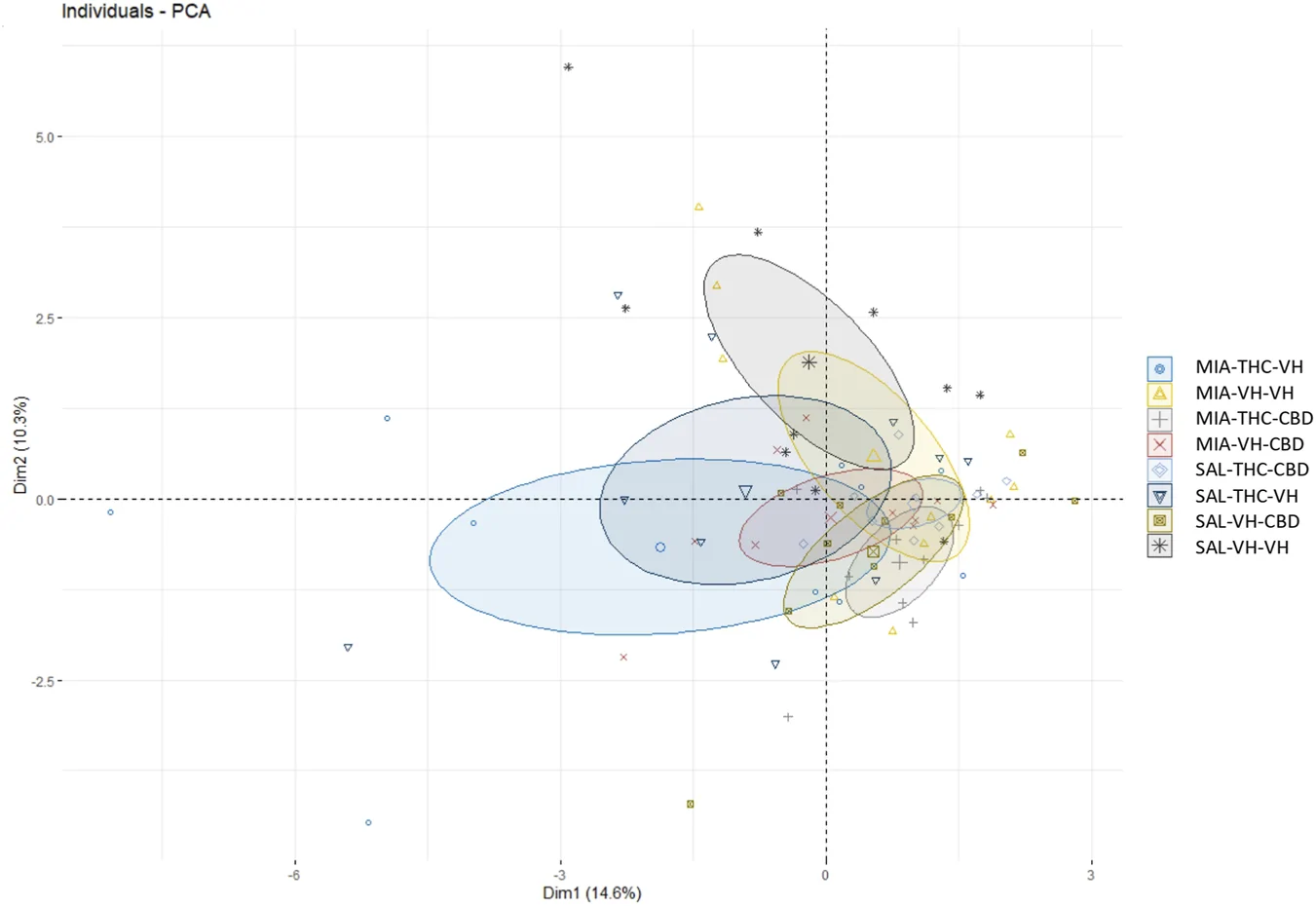
Principal Component Analysis (PCA) analysis graphical representation. PCA was performed on the gut microbiota data, accounting for 24.9% of the variance observed in microbial composition across the different groups.

Principal Component Analysis (PCA) (Figure 7) was performed to explore the overall structure and variability of the microbiota communities among the eight experimental animal groups. The PCA1 and PCA2 components explained 24.9% of the variance between the different animal groups. We found that THC-VH groups (both saline and MIA) showed the highest data dispersion compared to the other groups, indicating differences in the gut microbiota composition associated with THC treatment during adolescence. In addition, MIA-offspring receiving CBD showed a distribution of microbiota taxa more similar to that of the healthy group.

### 3.6 LDA of the microbiota

**FIGURE 8:**
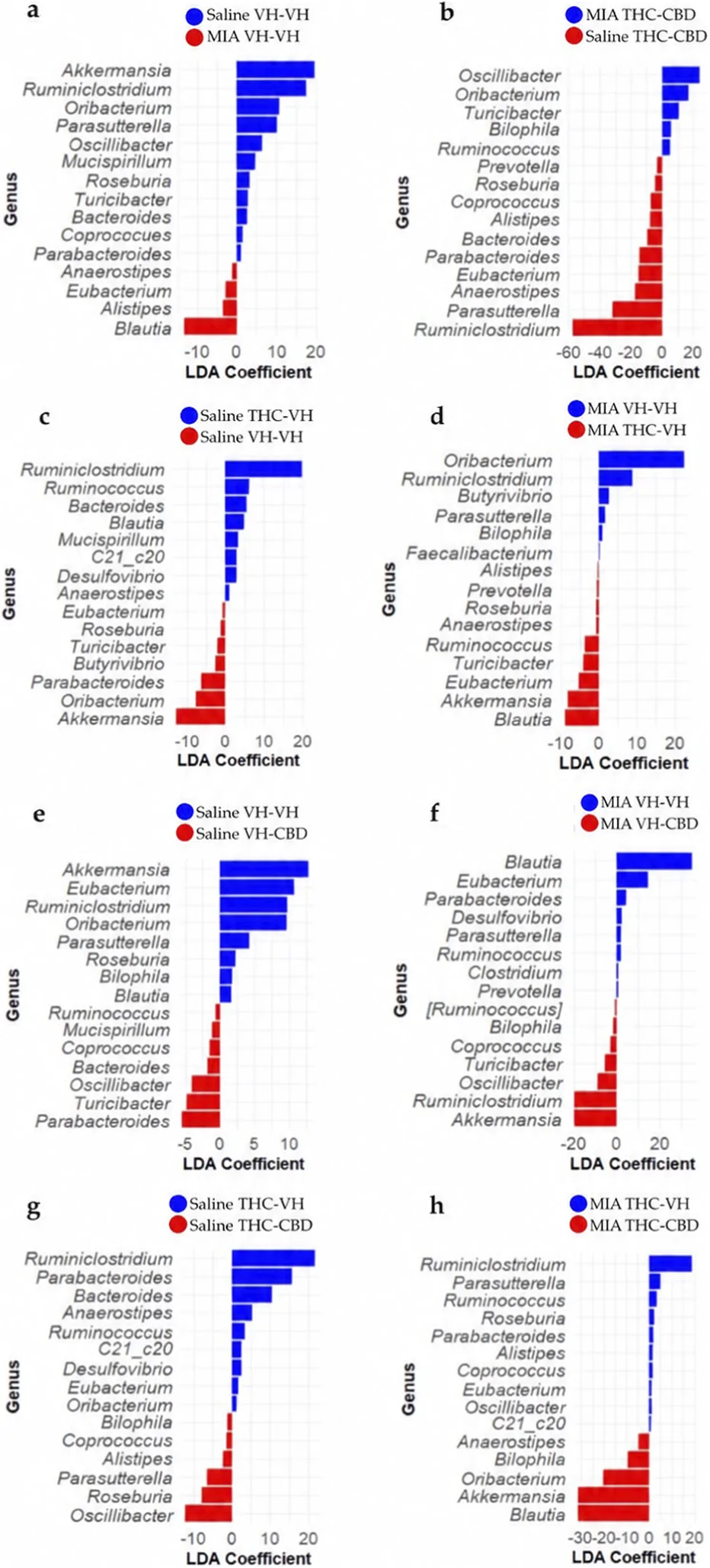
Each panel depicts a pairwise comparison between the indicated experimental groups: **(a)** MIA_VH_VH vs Saline_VH_VH; **(b)** MIA_THC_CBD vs Saline_THC_CBD; **(c)** Saline_THC_VH vs Saline_VH_VH; **(d)** MIA_THC_VH vs MIA_VH_VH; **(e)** Saline_VH_CBD vs Saline_VH_VH; **(f)** MIA_VH_CBD vs MIA_VH_VH; **(g)** Saline_THC_CBD vs Saline_THC_VH; **(h)** MIA_THC_CBD vs MIA_THC_VH. Blue bars represent taxa enriched in the first group, whereas red bars represent taxa enriched in the second group.

Linear discriminant analysis (LDA) identified distinct bacterial genera discriminating each group comparison. The main bacterial genera that best discriminate between pathological and healthy control group included *Akkermansia* and *Blautia*, with *Akkermansia* positively associated with saline animals (Figure 8a). In saline-offspring, THC treatment was characterized by *Ruminoclostridium*, whereas untreated animals were defined by *Akkermansia* (Figure 8c); in MIA offspring, *Oribacterium* and *Blautia* were the primary discriminating genera (Figure 8d). CBD treatment was associated with *Parabacteroides* in saline animals and *Akkermansia* in untreated controls (Figure 8e), whereas in MIA animals *Blautia* dominated untreated groups and *Akkermansia* in CBD groups (Figure 8f). Combined THC + CBD treatment in both Saline and MIA animals was characterized by *Ruminoclostridium* and *Oscillibacter* as the primary discriminating genera (Figures 8b, g, h).

### 3.7 Spearman correlations

Correlation analyses were performed within each experimental group to evaluate associations between gut microbial composition and the main phenotypic outcomes of the study.

#### 3.7.1 Correlation between gut microbiota taxa and brain ventricular volume

**FIGURE 9:**
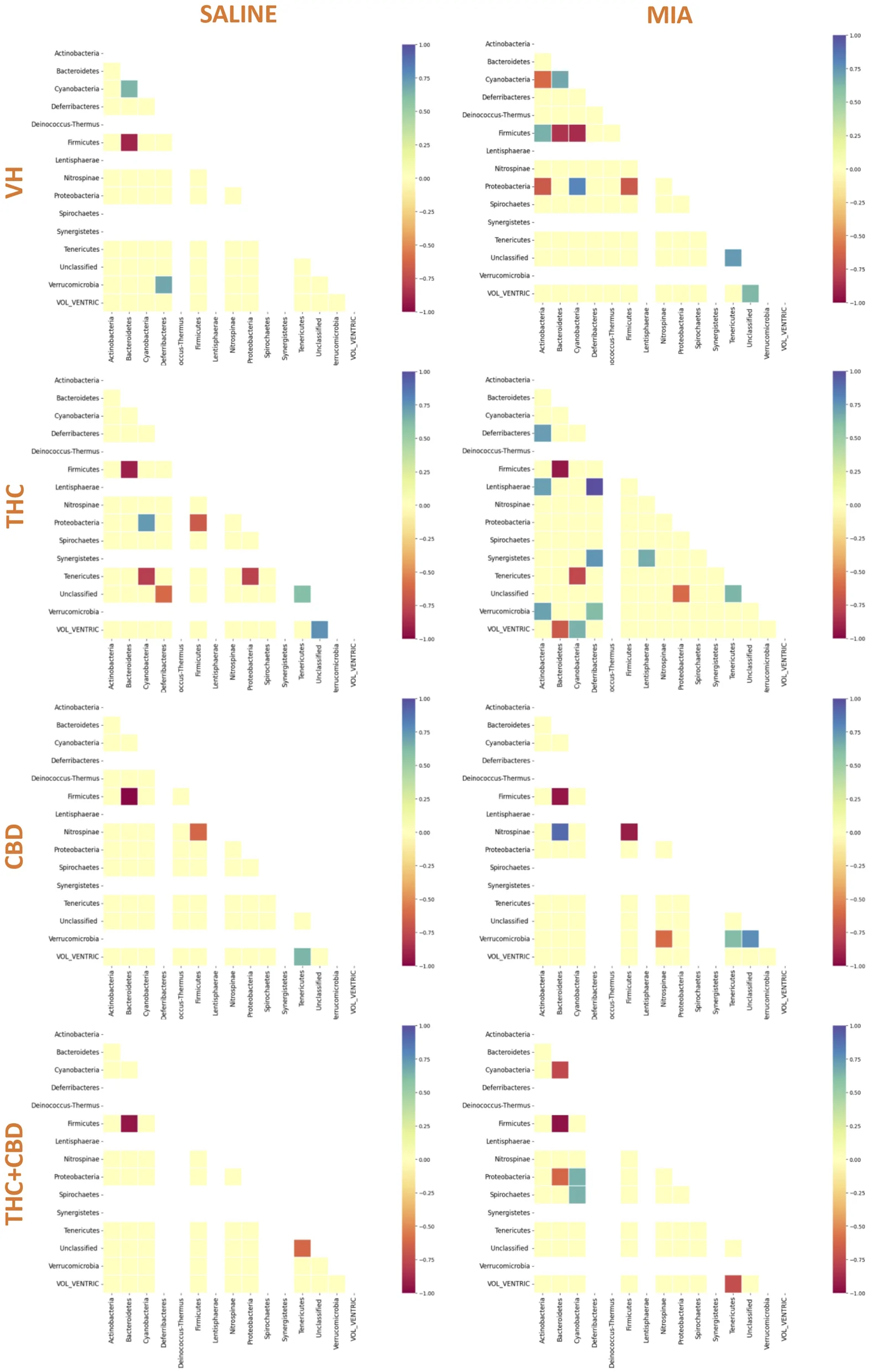
Spearman correlation heatmaps showing associations between gut microbiota phyla and ventricular volume across experimental conditions. Correlations were calculated separately for each group. Color gradients represent the direction and magnitude of the correlation coefficients, with positive correlations and negative associations indicated by opposing scales. Only statistically significant correlations are shown (p < 0.05).

No significant correlations were observed between ventricular volume and the microbial taxa specifically associated with the MIA condition (MIA-VH-VH group) (Figure 9). However, significant associations were identified between ventricular volume and microbial taxa modulated by THC and CBD treatment. Specifically, in MIA-offspring, THC-associated microbial changes were characterized by a significant negative correlation between ventricular volume and *Bacteroidota* (p < 0.05) and a positive trend for *Bacillota* (p = 0.08). For CBD, a significant positive correlation between ventricular volume and *Mycoplasmatota* (p < 0.05) was found in Saline-offspring. In contrast, MIA-offspring exhibited an opposite pattern, showing a negative correlation trend (p = 0.09), which became significant in the MIA-THC-CBD group (p < 0.05).

#### 3.7.2 Correlation between gut microbiota taxa and IOS in brain

**FIGURE 10:**
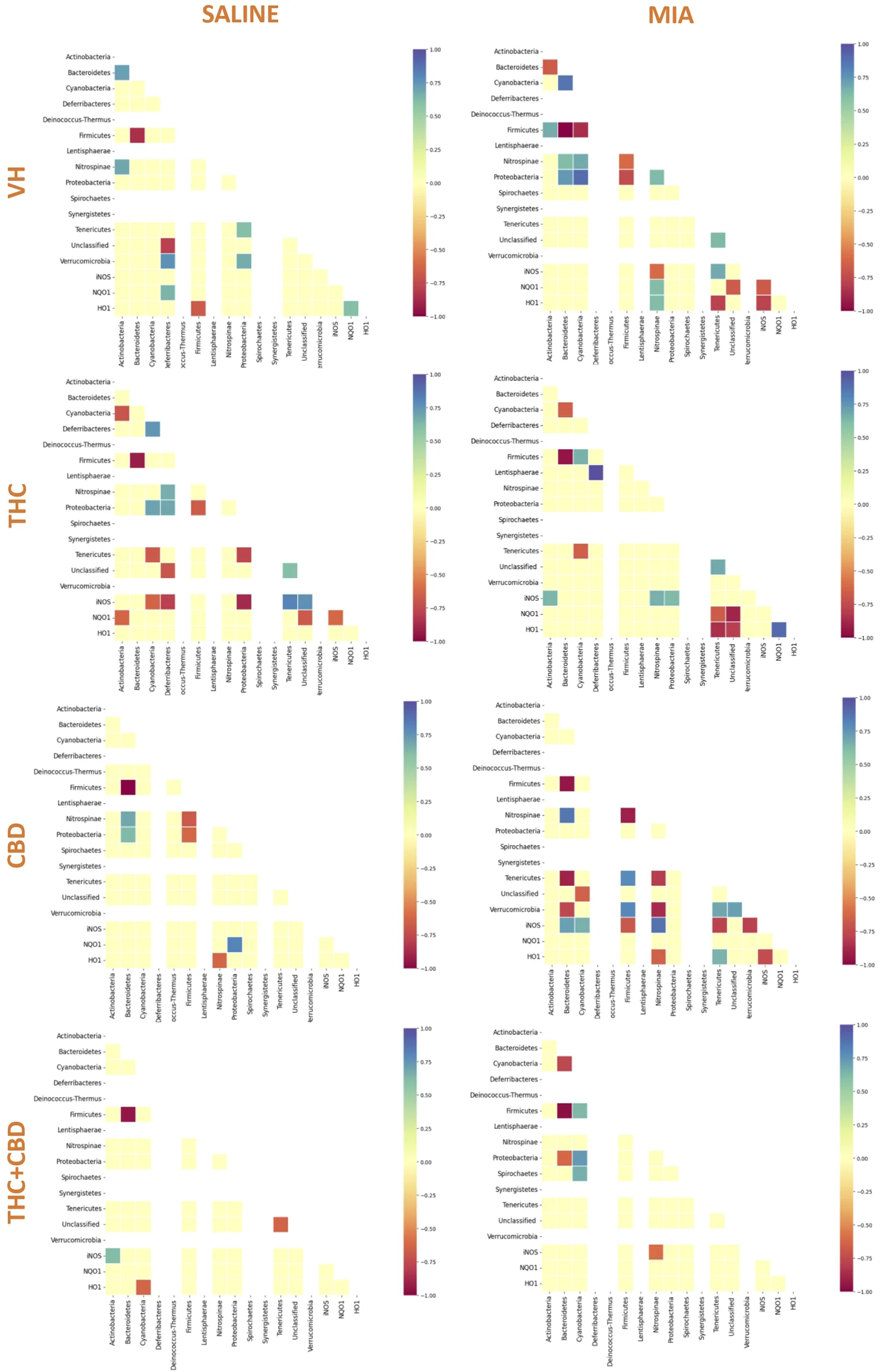
Spearman correlation heatmaps showing associations between gut microbiota phyla and prefrontal inflammatory/oxidative (IOS) markers across experimental groups. Correlations were calculated separately for each group. Heatmaps illustrate correlation coefficients between the most representative bacterial phyla and IOS markers (iNOS, NQO1 and HO1), allowing comparison of association patterns across treatments. Only statistically significant correlations are shown (p < 0.05).

Correlations between gut microbiota and oxidative and inflammatory stress markers in prefrontal cortex (iNOS, NQO1, HO1) revealed phenotype- and treatment-dependent associations (Figure 10). Under basal conditions, the MIA phenotype was characterized by a *Mycoplasmatota* profile associated with a trend toward increased iNOS levels (p = 0.09) and a significant reduction in HO1 (p < 0.05), suggesting a shift toward a pro-inflammatory state accompanied by a diminished antioxidant profile. After THC exposure, Saline-offspring showed a strong negative association with iNOS (p = 0.004), whereas *Mycoplasmatota* displayed a positive correlation with iNOS (p < 0.01). In MIA-offspring, *Mycoplasmatota* was negatively associated with antioxidant markers, including HO1 (p < 0.01) and NQO1 (p = 0.07). In contrast, under CBD treatment, MIA-offspring showed the strongest positive correlation between *Nitrospinota* (p < 0.005) and iNOS, accompanied by a positive association with *Bacteroidota* (p < 0.05). Conversely, *Mycoplasmatota* was negatively associated with iNOS (p < 0.05) but almost positively associated with HO1 (p = 0.08), suggesting differential relationships with inflammatory and antioxidant pathways. By comparison, Saline-offspring displayed relatively limited associations, the most notable being a positive correlation between *Pseudomonadota* and NQO1 (p < 0.01).

## 4 Discussion

The present study: 1) provided further evidence of the existence of gut microbiota alterations in the schizophrenia-like model induced by Poly I:C, including reduced microbial diversity, consistent with findings in patients with schizophrenia; and 2) demonstrated that exposure to cannabinoids during adolescence, particularly following MIA challenge, significantly alters gut microbiota composition and diversity in adulthood.

In this context, our findings are consistent with clinical studies reporting a dysbiotic gut microbiota profile with marked reduction in beneficial butyrate-producing bacteria, such as *Faecalibacterium* and *Roseburia* (Li Z. et al., 2024). In addition, MIA animals in the present study exhibited a reduced abundance of *Clostridium hiranonis* and a complete absence of *Akkermansia muciniphila*, two taxa closely associated with gut barrier integrity and anti-inflammatory activity. Such alterations in microbial populations are often associated with increased inflammation and metabolic imbalance, which may contribute to impaired immune regulation and abnormal neurodevelopmental outcomes (Tsamakis et al., 2022). Furthermore, the differential impact of cannabinoids observed in this study mirrors the distinct neurobiological profiles of THC and CBD. While THC has been shown to disrupt key neural circuits including reward pathways and exacerbate psychotic-like symptoms (Fu et al., 2021), CBD may exert anti-inflammatory actions and promote the restoration of gut barrier integrity (Tao et al., 2025), potentially counteracting some of the deleterious effects of the MIA challenge and THC treatment, though direct functional validation was beyond the scope of the present study.

The observed reduction in bacterial diversity in MIA-offspring is in line with our previous work showing a reduced number of species and relative abundance distribution (Romero-Miguel et al., 2023) and reflects features of gut dysbiosis linked to neurodevelopmental disorders (Hsiao et al., 2013). Also, the influence of CBD on microbial richness appears to be highly context-dependent, as it leads to a reduction in bacterial richness in healthy controls but not in MIA-offspring. However, combined exposure to THC and CBD under MIA conditions results in a further exacerbation of richness loss, indicating potential synergistic or additive effects of cannabinoids in the presence of immune-mediated developmental perturbations. These findings highlight the complex interplay between cannabinoid signaling, immune activation and neurodevelopmental disturbances in shaping gut microbiota composition. Reduced microbial diversity and altered community composition, as shown by the Shannon and Chao indices, are indicative of reduced functional capacity of the microbial ecosystem, which could contribute to dysregulated gut-brain axis signaling and increased vulnerability to neuropsychiatric disorders later in life (Guo et al., 2025).

Our results demonstrate that, at the phylum level, *Bacillota* and *Bacteroidota* dominated the gut microbiota ecosystem across all experimental conditions, consistent with their established role as major constituents of the mammalian gut microbiota. Given their importance in maintaining microbial homeostasis, we evaluated the *Bacillota/Bacteroidota* (B/B) ratio, a commonly used indicator of gut microbial balance whose elevation is frequently been associated with neuropsychiatric disorders. Although some clinical studies in schizophrenia have reported an elevated B/B ratio, our analysis did not reveal a significant effect of the MIA model on this parameter, showing only a non-significant trend toward increase, nor any interaction between MIA and cannabinoids (Szeligowski et al., 2020; Xiang et al., 2022). This lack of effect is consistent with previous findings in prenatal immune activation models (Juckel et al., 2021; Romero-Miguel et al., 2023). In contrast, CBD treatment reduced the B/B ratio in both saline and MIA-offspring, whereas THC tended to increase it, suggesting that cannabinoids can modulate overall microbial community structure independently of the underlying pathological condition (Ibrahim et al., 2022; Chen et al., 2025).

Within the phylum *Bacillota*, we observed significant modulations in families such as Clostridiaceae and *Clostridiales Family XVI*, the latter being notably increased by combined THC and CBD treatment in Saline-offspring. These trends were largely preserved at the genus and species levels, where THC selectively enriched *Clostridium hiranonis*, *Roseburia* and *Oscillibacter* in MIA-offspring. These microorganisms play an important role (directly or indirectly) in short-chain fatty acid (SCFA) production and energy metabolism (Zhang et al., 2022). They are also prolific butyrate producers (Li et al., 2022; Ye et al., 2023), conferring antioxidant and anti-inflammatory effects through mechanisms such as suppression of lipid peroxidation (MDA) (Vincent et al., 2013) and inhibition of NF-κB-dependent transcription of TNF-α and IL-6 (Arpaia and Rudensky, 2014; Kristiansson et al., 2021). Similarly, *Oscillospiraceae* abundance increased following CBD treatment in MIA-offspring. Given the well-established anti-inflammatory properties of this family, which is largely attributed to the production of SCFA, particularly butyrate production, this finding may reflect a microbiota-mediated mechanism through which CBD contributes to the restoration of this taxon in MIA-offspring.

Specific taxonomic shifts were also observed at the family level within the *Bacteroidota* phylum, which is fundamental for immune homeostasis and polysaccharide metabolism (Yoo et al., 2020; Afzaal et al., 2022; Kim et al., 2024). For instance, CBD treatment increased the relative abundance of *Prevotellaceae* in both Saline and MIA-offspring, whereas THC treatment showed the opposite pattern. This CBD-induced shift has previously been linked to reduced SCFA production and increased oxidative stress in rodent models of neuroinflammation (Mostafavi Abdolmaleky and Zhou, 2024; Graf et al., 2025). *Prevotellaceae* is commonly found in the human gut microbiota and is typically associated with diets rich in complex carbohydrates and fibre. However, while clinical reports on *Prevotellaceae* in schizophrenia remain heterogeneous (Su et al., 2025; Tang et al., 2025), our data suggest that cannabinoids can differentially modulate this family. Moreover, CBD also induced a marked upregulation of *Flavobacteriaceae,* a taxon presumed to produce enzymes involved in oxidative regulation and maintenance of redox balance (Zhou et al., 2024b), which may contribute to a healthier microbial profile, as its increase has been associated with symptom remission in schizophrenia (Romero-Miguel et al., 2023).

At the species level, THC enriched *Parabacteroides distasonis* in MIA-offspring, a taxon associated with immune regulation via aryl-hydrocarbon receptor activation, a pathway implicated in the attenuation of oxidative stress (Abreu and Abreu et al., 2021). In contrast, CBD, either alone or in combination with THC, reduced *Parabacteroides* and *Parabacteroides distasonis* abundance, suggesting a shift toward a microbial profile with potentially diminished anti-inflammatory capacity. These findings underscore the distinct and cannabinoid-specific effects of THC and CBD on gut microbial composition. Furthermore, *Akkermansia muciniphila* emerged as an important contributor to group discrimination in the multivariate analyses. This observation is particularly relevant, as *A. muciniphila* is essential for maintaining gut barrier integrity. Its absence in untreated MIA-offspring reflects an increased susceptibility to gut barrier dysfunction and inflammation, whereas the partial restoration observed following cannabinoid exposure suggests these compounds might help mitigate some of the microbiota alterations associated with the MIA phenotype (Khalili et al., 2024).

Beyond the dominant phyla, significant modulations were also observed in less abundant taxa, including *Pseudomonadota*, *Nitrospinota* and *Deferribacterota*. Both CBD and THC increased the relative abundance of *Nitrospinota*, a phylum involved in nitrogen cycling, suggesting a cannabinoid-driven modulation of microbial nitrogen metabolism that is independent of early immune activation (Lücker et al., 2010; Daims et al., 2015). In contrast, *Deferribacterota* abundance was notably reduced in MIA-offspring and was undetectable in animals exposed to either THC and CBD alone, suggesting that this phylum is highly sensitive to early-life inflammatory insults and cannabinoid exposure. Interestingly, its persistence in animals receiving the combined THC + CBD treatment may indicate a differential response to co-administration of both cannabinoids, highlighting a potentially distinct modulatory effect of the combined treatment on this microbial taxon (Rooks and Garrett, 2016; Borrego-Ruiz and Borrego, 2025).

Within the phylum *Pseudomonadota*, cannabinoid exposure appeared to limit the expansion of potentially opportunistic taxa such as *Enterobacteriaceae* and *Pasteurellaceae,* particularly in MIA-offspring. This effect is consistent with previous evidence indicating that SCFA-rich environments can suppress the growth of *Pseudomonadota* taxa members and mitigate the oxidative and inflammatory burden associated with their expansion (Sun et al., 2024). These microbial shifts may have important functional consequences. For instance, cannabinoid treatment decreased the abundance of *Bilophila*, a genus frequently elevated in patients with schizophrenia and linked to low-grade inflammation, gut dysfunction and cognitive impairment (Su et al., 2025; Ataei et al., 2026). Given that *Bilophila* has been shown to negatively impact butyrate-producing bacterial populations, its reduction, together with the enrichment of beneficial taxa, supports a shift toward a more anti-inflammatory and metabolically favorable gut environment. Collectively, these microbiota changes may be contributing to the attenuation of the MIA-induced inflammatory alterations by promoting a more balanced and protective intestinal ecosystem.

Finally, correlation analyses linking gut microbiota composition with ventricular volume and prefrontal cortex oxidative/inflammatory markers further support the taxonomic shifts described above. These associations are consistent with previous evidence showing that schizophrenia- and MIA-related phenotypes are accompanied by gut dysbiosis, immune activation, and altered redox balance, and that microbial alterations may contribute to neuroinflammatory processes and structural brain changes in vulnerable offspring (Nguyen et al., 2021; Zhou et al., 2024a). In relation to the MIA phenotype, *Mycoplasmatota* was associated with increased iNOS and reduced HO1 levels, a pattern consistent with the pro-inflammatory and impaired antioxidant status reported in schizophrenia and MIA models (Su et al., 2025). These findings align with the notion that MIA-induced dysbiosis may contribute to neuroinflammation while weakening antioxidant defence, thereby contributing to neurobiological alterations, including ventricular enlargement (Zhou et al., 2024a). THC exposure showed the most prominent microbiota-phenotype associations. In MIA-offspring, THC-related microbial changes were associated with ventricular volume, with positive correlations involving *Bacillota* and a negative correlation with *Bacteroidota*. In Saline-offspring, the anti-inflammatory *Mycoplasmatota* taxa was positively associated with iNOS, whereas pro-inflammatory taxa, such as *Pasteurellaceae* and *Desulfovibrionaceae*, showed negative associations. Rather than reflecting a uniform anti-inflammatory effect, this pattern suggests that THC reshapes specific host-microbiota relationships in a phenotype-dependent manner, consistent with previous reports showing that cannabinoid effects on the gut microbiota depend on the underlying inflammatory status (Ibrahim et al., 2022). Moreover, the negative associations between *Mycoplasmatota* and the antioxidant markers HO1 and NQO1 in MIA-offspring, further support its potential role as an indicator of inflammatory burden and impaired redox regulation (Su et al., 2025). Meanwhile, CBD treatment revealed a different pattern of associations. The relationship between *Mycoplasmatota* and ventricular volume varied according to the phenotype, being positive in Saline-offspring but negative in MIA-offspring, even in the presence of THC (MIA-THC-CBD group). In addition, in MIA-offspring, *Nitrospinota* and *Bacteroidota* were positively correlation with iNOS, whereas *Mycoplasmatota* showed a negative correlation with iNOS but a positive association with HO1. These findings suggest that CBD may differentially modulate inflammatory and antioxidant pathways, potentially promoting a reorganization of microbiota-host interactions distinct from that induced by THC (Ibrahim et al., 2022; Lamanna-Rama et al., 2024). Collectively, our results show that THC and CBD differentially modulate the gut-brain axis in a phenotype-dependent manner. While THC-induced enrichment of specific *Bacillota* taxa may counteract some MIA-associated inflammatory alterations, CBD appears to engage distinct inflammatory and antioxidant signaling pathways. Overall, these observations extend prior work by demonstrating that cannabinoid-induced microbiota changes are not uniform but depend on the neurodevelopmental background, and are linked to both structural and redox-related brain outcomes (Nguyen et al., 2021; Lamanna-Rama et al., 2024). They also highlight the complexity of cannabinoid-microbiota interactions and their potential relevance for neurodevelopmental disorders characterized by immune dysregulation and altered neuroinflammatory signaling.

### 4.1 Limitations

Several limitations should be acknowledged when interpreting these findings. First, although the sample size was sufficient to detect major effects on gut microbiota composition, subtle effects may have gone undetected, highlighting the need for replication in larger cohorts. Second, despite its extensive validation, the MIA model cannot fully recapitulate the complexity of human neuropsychiatric disorders, and species-specific physiological and dietary differences between rodents and humans warrant caution when extrapolating these findings to humans. Moreover, prenatal PolyI:C exposure increases the risk of schizophrenia-like alterations in offspring, but not all animals developed the phenotype to the same extent. Consequently, animals may differ in the severity of behavioural and neurobiological abnormalities, as well as in their associated microbiota profiles, which may partly explain the differences in dysbiotic signatures observed between cohorts. Third, while analyses of microbial richness and diversity were hypothesis-driven, taxon-specific findings and their associations with brain structural and biochemical measures remain largely exploratory. Consequently, the reported correlations should be interpreted as associative rather than causal. Fourth, only male animals were included, as females do not exhibit the characteristic schizophrenia-like phenotype observed in males at PND100 (Casquero-Veiga et al., 2023). Given growing evidence for sex-dependent differences in microbiota-immune interactions in MIA models (Tartaglione et al., 2022; Salia et al., 2025). Future studies should incorporate both sexes. Finally, microbiota analysis was based on relative abundance from 16S rRNA sequencing, without absolute quantification of total bacterial counts, preventing assessment of potential changes in total microbial biomass and their functional significance.

## 5 Conclusion

Collectively, our findings suggest that adolescent THC and CBD exposure, exert distinct modulatory effects on gut microbiota composition and its relationship with brain structure and oxidative/inflammatory status in the MIA model. THC displayed a dual profile, promoting the enrichment of taxa associated with SCFA-production and antioxidant capacity while also increasing microbial signatures linked to pro-inflammatory states, suggesting both beneficial and potentially adverse effects depending on the neurodevelopmental context. In contrast, CBD was associated primarily with an enrichment of taxa with potential SCFA-producing and mucin-associated functions, along with a reduction in certain pro-inflammatory genera. Moreover, the correlation patterns observed between microbial taxa, ventricular volume and oxidative/inflammatory markers were consistent with a partial normalization of gut–brain axis signaling in the MIA-offspring. Together, these findings support the existence of a microbiota-mediated pathways through which cannabinoids may influence neuroinflammatory/oxidant processes and schizophrenia-like phenotypes. Although exploratory and not indicative of causality, our results highlight the gut microbiota-endocannabinoid axis as a potential therapeutic target in neurodevelopmental disorders, warranting deeper research into its mechanistic and translational relevance.

## Data Availability

The raw sequences obtained from all animals were deposited in NCBI SRA under BioProject ID: PRJNA1484513.
