# Supplementary material for: THC and CBD differentially modulated gut microbiota in the Poly I:C rat model of schizophrenia
Source: Front Pharmacol. 2026 Aug 10;17:1858604. doi: 10.3389/fphar.2026.1858604 (PMC13500884; doi:10.3389/fphar.2026.1858604)
Supplement: Supplementary file 1 [file Table1.docx]

**Supplementary Table 1.** **Data from phyla populations**. Table shows the mean ± SEM for each group, the p-value from the Kruskal-Wallis (KW) analysis, and the FDR-adjusted q-value obtained by the Benjamini-Hochberg procedure. Bold and underlined values indicate statistical significance: *p < 0.05; **p < 0.01; ***p < 0.001. For FDR, q < 0.05 is considered significant.

| Phylum | Sal  VH-VH | | MIA  VH-VH | | Sal  THC-VH | | MIA  THC-VH | | Sal  VH-CBD | | MIA  VH-CBD | | Sal  THC-CBD | | MIA  THC-CBD | | KW  (p- value) | FDR (q-value) | η² |
| --- | --- | --- | --- | --- | --- | --- | --- | --- | --- | --- | --- | --- | --- | --- | --- | --- | --- | --- | --- |
|  | **Mean** | **SEM** | **Mean** | **SEM** | **Mean** | **SEM** | **Mean** | **SEM** | **Mean** | **SEM** | **Mean** | **SEM** | **Mean** | **SEM** | **Mean** | **SEM** |  |  |  |
| Actinomycetota | 0.016 | 0.006 | 0.142 | 0.071 | 0.042 | 0.021 | 0.223 | 0.127 | 0.05 | 0.021 | 0.05 | 0.040 | 0.046 | 0.018 | 0.058 | 0.058 | 0.386 | 0.540 | 0.094 |
| Bacteroidota | 35.824 | 3.444 | 30.846 | 2.496 | 27.628 | 1.496 | 32.485 | 2.132 | 34.672 | 4.063 | 36.574 | 1.904 | 33.797 | 1.204 | 32.233 | 2.450 | **0.059** | 0.165 | 0.172 |
| Deferribacterota | 0.217 | 0.142 | 0 | 0.000 | 0.109 | 0.068 | 0.048 | 0.034 | 0 | 0.000 | 0 | 0.000 | 0 | 0.000 | 0 | 0.000 | **0.001** | **0.014** | 0.314 |
| Deinococcota | 0 | 0.000 | 0.006 | 0.006 | 0 | 0.000 | 0 | 0.000 | 0.007 | 0.007 | 0 | 0.000 | 0 | 0.000 | 0 | 0.000 | 0.531 | 0.619 | 0.077 |
| Bacillota | 57.928 | 3.545 | 62.405 | 3.139 | 67.284 | 2.070 | 64.272 | 2.352 | 62.338 | 4.180 | 60.602 | 1.890 | 62.668 | 1.247 | 64.989 | 2.275 | 0.161 | 0.281 | 0.133 |
| Lentisphaerota | 0 | 0.000 | 0.045 | 0.045 | 0 | 0.000 | 0.002 | 0.001 | 0 | 0.000 | 0 | 0.000 | 0 | 0.000 | 0 | 0.000 | 0.164 | 0.255 | 0.132 |
| Nitrospinota | 0.008 | 0.008 | 0.007 | 0.007 | 0.002 | 0.002 | 0.067 | 0.030 | 0.037 | 0.021 | 0.104 | 0.034 | 0.036 | 0.036 | 0.045 | 0.025 | **0.003** | **0.021** | 0.270 |
| Pseudomonadota | 4.019 | 0.798 | 4.035 | 0.988 | 3.859 | 1.199 | 1.936 | 0.530 | 2.128 | 0.318 | 1.43 | 0.207 | 1.395 | 0.191 | 2.02 | 0.460 | **0.020** | 0.070 | 0.210 |
| Spirochaetota | 0 | 0.000 | 0.005 | 0.005 | 0.004 | 0.004 | 0.007 | 0.007 | 0.006 | 0.006 | 0 | 0.000 | 0 | 0.000 | 0.017 | 0.017 | 0.869 | 0.9358 | 0.040 |
| Synergistetota | 0 | 0.000 | 0 | 0.000 | 0 | 0.000 | 0.001 | 0.001 | 0 | 0.000 | 0 | 0.000 | 0 | 0.000 | 0 | 0.000 | 0.429 | 0.5460 | 0.089 |
| Mycoplasmatota | 1.419 | 0.522 | 2.167 | 0.552 | 0.789 | 0.236 | 0.627 | 0.265 | 0.424 | 0.237 | 0.82 | 0.248 | 1.255 | 0.546 | 0.542 | 0.134 | 0.067 | 0.1563 | 0.168 |
| unclassified bacteria | 0.332 | 0.195 | 0.227 | 0.080 | 0.234 | 0.105 | 0.227 | 0.122 | 0.254 | 0.144 | 0.304 | 0.138 | 0.714 | 0.213 | 0.029 | 0.029 | 0.075 | 0.1500 | 0.163 |
| Verrucomicrobiota | 0.072 | 0.043 | 0 | 0.000 | 0 | 0.000 | 0.069 | 0.038 | 0 | 0.000 | 0.099 | 0.053 | 0.032 | 0.032 | 0 | 0.000 | **0.006** | **0.028** | 0.250 |
