# Supplementary material for: THC and CBD differentially modulated gut microbiota in the Poly I:C rat model of schizophrenia
Source: Front Pharmacol. 2026 Aug 10;17:1858604. doi: 10.3389/fphar.2026.1858604 (PMC13500884; doi:10.3389/fphar.2026.1858604)
Supplement: Supplementary file 2 [file Table2.docx]

| Family  **Supplementary Table 2.** **Data from family populations**. Table shows mean ± SEM per group. Statistical comparisons were performed using Kruskal-Wallis (KW) or 3-way ANOVA as appropriate. FDR-adjusted q-values were calculated using the Benjamini-Hochberg procedure. *p < 0.05; **p < 0.01; ***p < 0.001. Effect size is expressed as η². | Sal  VH-VH | | MIA  VH-VH | | Sal  THC-VH | | MIA  THC-VH | | Sal  VH-CBD | | MIA  VH-CBD | | | Sal  THC-CBD | | MIA  THC-CBD | | KW  (p- value) | FDR (q-value) | 3-way ANOVA  (p- value) | η² |
| --- | --- | --- | --- | --- | --- | --- | --- | --- | --- | --- | --- | --- | --- | --- | --- | --- | --- | --- | --- | --- | --- |
|  | **Mean** | **SEM** | **Mean** | **SEM** | **Mean** | **SEM** | **Mean** | **SEM** | **Mean** | **SEM** | | **Mean** | **SEM** | **Mean** | **SEM** | **Mean** | **SEM** |  |  |  |  |
| *Acholeplasmataceae* | 1,243 | 0,483 | 1,879 | 0,557 | 0,645 | 0,245 | 0,506 | 0,270 | 0,379 | 0,238 | | 0,783 | 0,251 | 1,092 | 0,493 | 0,443 | 0,138 | 0.199 | 0.497 | - | 0.124 |
| *Anaeroplasmataceae* | 0,070 | 0,067 | 0,145 | 0,104 | 0,139 | 0,063 | 0,004 | 0,003 | 0,039 | 0,026 | | 0,017 | 0,017 | 0,109 | 0,083 | 0,066 | 0,042 | 0.835 | 0.907 | **-** | 0.044 |
| *Bacillaceae* | 0,206 | 0,102 | 0,122 | 0,072 | 0,364 | 0,146 | 0,218 | 0,120 | 0,093 | 0,059 | | 0,303 | 0,265 | 0,046 | 0,024 | 0,091 | 0,056 | 0.359 | 0.667 | - | 0.098 |
| *Bacteroidaceae* | 8,008 | 1,142 | 5,594 | 0,807 | 4,597 | 0,442 | 5,594 | 0,968 | 4,537 | 0,947 | | 5,894 | 0,851 | 6,519 | 0,783 | 5,986 | 0,575 | 0.071 | 0.236 | - | 0.165 |
| *Catabacteriaceae* | 0,000 | 0,000 | 0,041 | 0,037 | 0,040 | 0,026 | 0,006 | 0,005 | 0,016 | 0,016 | | 0,012 | 0,007 | 0,004 | 0,004 | 0,057 | 0,040 | 0.667 | 0.840 | - | 0.063 |
| *Clostridiaceae* | 11,143 | 1,160 | 9,090 | 0,799 | 13,351 | 1,056 | 10,885 | 0,957 | 7,748 | 1,126 | | 9,712 | 0,977 | 9,043 | 0,723 | 9,253 | 1,278 | **0.041** | 0.179 | - | 0.185 |
| *Clostridiales Family XI. Incertae Sedis* | 0,089 | 0,063 | 0,095 | 0,066 | 0,043 | 0,041 | 0,110 | 0,042 | 0,017 | 0,017 | | 0,095 | 0,062 | 0,109 | 0,071 | 0,000 | 0,000 | **0.045** | 0.187 | - | 0.182 |
| *Clostridiales Family XIII. Incertae Sedis* | 0,165 | 0,115 | 0,105 | 0,105 | 0,071 | 0,048 | 0,034 | 0,034 | 0,000 | 0,000 | | 0,000 | 0,000 | 0,047 | 0,047 | 0,000 | 0,000 | 0.279 | 0.572 | - | 0.109 |
| *Clostridiales Family XVI. Incertae Sedis* | 0,074 | 0,064 | 0,179 | 0,061 | 0,163 | 0,061 | 0,129 | 0,033 | 0,017 | 0,011 | | 0,101 | 0,032 | 0,249 | 0,051 | 0,103 | 0,030 | **0.006** | **0.031** | - | 0.251 |
| *Coriobacteriaceae* | 0,009 | 0,005 | 0,142 | 0,071 | 0,026 | 0,017 | 0,161 | 0,088 | 0,021 | 0,011 | | 0,036 | 0,029 | 0,026 | 0,015 | 0,058 | 0,058 | 0.461 | 0.750 | - | 0.085 |
| *Cyclobacteriaceae* | 0,068 | 0,020 | 0,057 | 0,021 | 0,036 | 0,013 | 0,031 | 0,011 | 0,086 | 0,042 | | 0,058 | 0,025 | 0,012 | 0,009 | 0,077 | 0,034 | 0.430 | 0.722 | - | 0.088 |
| *Cytophagaceae* | 2,120 | 0,704 | 0,909 | 0,165 | 1,085 | 0,139 | 0,975 | 0,186 | 0,787 | 0,291 | | 0,857 | 0,181 | 0,734 | 0,186 | 1,229 | 0,216 | 0.266 | 0.558 | - | 0.112 |
| *Deferribacteraceae* | 0,217 | 0,142 | 0,000 | 0,000 | 0,109 | 0,068 | 0,048 | 0,034 | 0,000 | 0,000 | | 0,000 | 0,000 | 0,000 | 0,000 | 0,000 | 0,000 | **0.0008** | **0.008** | - | 0.314 |
| *Desulfobacteraceae* | 0,021 | 0,021 | 0,229 | 0,229 | 0,000 | 0,000 | 0,012 | 0,012 | 0,000 | 0,000 | | 0,000 | 0,000 | 0,000 | 0,000 | 0,000 | 0,000 | **0.**644 | 0.824 | - | 0.065 |
| *Desulfovibrionaceae* | 1,252 | 0,308 | 2,080 | 0,782 | 1,608 | 0,548 | 0,967 | 0,425 | 0,550 | 0,222 | | 0,396 | 0,130 | 0,364 | 0,097 | 1,421 | 0,496 | 0.108 | 0.311 | - | 0.149 |
| *Desulfuromonadaceae* | 0,923 | 0,700 | 0,151 | 0,131 | 0,039 | 0,024 | 0,052 | 0,037 | 0,000 | 0,000 | | 0,012 | 0,009 | 0,077 | 0,071 | 0,000 | 0,000 | 0.154 | 0.404 | - | 0.135 |
| *Enterobacteriaceae* | 0,467 | 0,250 | 0,354 | 0,117 | 0,964 | 0,444 | 0,075 | 0,030 | 0,169 | 0,073 | | 0,055 | 0,030 | 0,326 | 0,197 | 0,054 | 0,027 | **0.0004** | **0.007** | - | 0.333 |
| *Entomoplasmataceae* | 0,046 | 0,030 | 0,022 | 0,022 | 0,001 | 0,001 | 0,104 | 0,044 | 0,000 | 0,000 | | 0,000 | 0,000 | 0,050 | 0,037 | 0,008 | 0,008 | **0.001** | **0.009** | - | 0.295 |
| *Erysipelotrichaceae* | 1,942 | 0,371 | 1,480 | 0,383 | 0,760 | 0,160 | 2,829 | 0,603 | 1,346 | 0,475 | | 1,862 | 0,321 | 1,680 | 0,324 | 0,842 | 0,197 | **0.032** | 0.155 | - | 0.194 |
| *Eubacteriaceae* | 2,069 | 0,487 | 1,373 | 0,168 | 1,581 | 0,308 | 1,872 | 0,166 | 1,702 | 0,246 | | 1,940 | 0,216 | 1,527 | 0,112 | 1,960 | 0,266 | - | - | Phe x CBD 0.134  Phe x THC 0.156 | 0.075 |
| *Flammeovirgaceae* | 0,051 | 0,028 | 0,069 | 0,046 | 0,042 | 0,024 | 0,007 | 0,007 | 0,024 | 0,020 | | 0,035 | 0,035 | 0,056 | 0,047 | 0,010 | 0,007 | 0.649 | 0.824 | - | 0.065 |
| *Flavobacteriaceae* | 0,140 | 0,048 | 0,279 | 0,071 | 0,264 | 0,093 | 0,306 | 0,142 | 0,958 | 0,358 | | 1,085 | 0,283 | 0,426 | 0,107 | 0,934 | 0,185 | **0.0002** | **0.010** | - | 0.362 |
| *Geobacteraceae* | 0,052 | 0,034 | 0,035 | 0,030 | 0,008 | 0,005 | 0,018 | 0,012 | 0,023 | 0,016 | | 0,019 | 0,010 | 0,028 | 0,020 | 0,100 | 0,061 | 0.985 | 0.985 | - | 0.018 |
| *Gracilibacteraceae* | 0,050 | 0,023 | 0,023 | 0,013 | 0,005 | 0,005 | 0,055 | 0,027 | 0,000 | 0,000 | | 0,008 | 0,008 | 0,027 | 0,027 | 0,000 | 0,000 | 0.059 | 0.212 | - | 0.172 |
| *Haloplasmataceae* | 0,332 | 0,195 | 0,227 | 0,080 | 0,234 | 0,105 | 0,227 | 0,122 | 0,254 | 0,144 | | 0,304 | 0,138 | 0,714 | 0,213 | 0,029 | 0,029 | 0.075 | 0.243 | - | 0.163 |
| *Holosporaceae* | 0,050 | 0,030 | 0,050 | 0,021 | 0,067 | 0,021 | 0,127 | 0,068 | 0,083 | 0,048 | | 0,137 | 0,055 | 0,037 | 0,025 | 0,034 | 0,017 | 0.401 | 0.699 | - | 0.092 |
| *Hyphomicrobiaceae* | 0,039 | 0,026 | 0,019 | 0,010 | 0,020 | 0,012 | 0,031 | 0,030 | 0,028 | 0,019 | | 0,061 | 0,041 | 0,006 | 0,006 | 0,050 | 0,021 | 0.811 | 0.897 | - | 0.047 |
| *Kopriimonadaceae* | 0,100 | 0,065 | 0,115 | 0,045 | 0,017 | 0,009 | 0,059 | 0,026 | 0,065 | 0,065 | | 0,054 | 0,026 | 0,023 | 0,015 | 0,014 | 0,009 | 0.249 | 0.540 | - | 0.115 |
| *Lachnospiraceae* | 25,420 | 3,099 | 28,286 | 1,833 | 32,462 | 3,159 | 28,464 | 1,854 | 27,037 | 3,378 | | 28,773 | 1,926 | 28,465 | 2,248 | 32,393 | 1,642 | 0.183 | 0.458 | - | 0.128 |
| *Lactobacillaceae* | 4,426 | 0,379 | 5,654 | 1,206 | 5,503 | 1,595 | 8,162 | 1,821 | 16,295 | 9,032 | | 7,595 | 0,967 | 7,740 | 2,315 | 6,319 | 1,915 | 0.302 | 0.602 | - | 0.106 |
| *Mycoplasmataceae* | 0,060 | 0,048 | 0,121 | 0,085 | 0,004 | 0,004 | 0,013 | 0,010 | 0,006 | 0,006 | | 0,020 | 0,018 | 0,004 | 0,004 | 0,025 | 0,011 | 0.416 | 0.711 | - | 0.090 |
| *Nitrospinaceae* | 0,008 | 0,008 | 0,007 | 0,007 | 0,002 | 0,002 | 0,067 | 0,030 | 0,037 | 0,021 | | 0,104 | 0,034 | 0,036 | 0,036 | 0,045 | 0,025 | **0.003** | **0.019** | - | 0.270 |
| *Nostocaceae* | 0,170 | 0,114 | 0,105 | 0,051 | 0,047 | 0,020 | 0,059 | 0,045 | 0,047 | 0,036 | | 0,011 | 0,007 | 0,058 | 0,036 | 0,054 | 0,036 | 0.902 | 0.953 | - | 0.035 |
| *Oscillospiraceae* | 0,729 | 0,243 | 0,772 | 0,160 | 0,739 | 0,164 | 1,129 | 0,335 | 1,197 | 0,250 | | 1,385 | 0,167 | 0,720 | 0,113 | 1,877 | 0,246 | **0.002** | **0.015** | - | 0.282 |
| *Oxalobacteraceae* | 0,115 | 0,050 | 0,207 | 0,075 | 0,034 | 0,016 | 0,136 | 0,065 | 0,568 | 0,144 | | 0,125 | 0,053 | 0,052 | 0,028 | 0,095 | 0,049 | **0.027** | 0.136 | - | 0.199 |
| *Paenibacillaceae* | 0,008 | 0,005 | 0,056 | 0,035 | 0,003 | 0,003 | 0,024 | 0,013 | 0,057 | 0,036 | | 0,047 | 0,018 | 0,063 | 0,030 | 0,025 | 0,020 | 0.515 | 0.797 | - | 0.079 |
| *Parvularculaceae* | 0,049 | 0,031 | 0,022 | 0,010 | 0,021 | 0,011 | 0,053 | 0,017 | 0,040 | 0,029 | | 0,083 | 0,026 | 0,020 | 0,012 | 0,013 | 0,009 | 0.152 | 0.397 | - | 0.135 |
| *Pasteurellaceae* | 0,408 | 0,193 | 0,450 | 0,310 | 0,667 | 0,289 | 0,068 | 0,039 | 0,231 | 0,102 | | 0,031 | 0,026 | 0,079 | 0,039 | 0,005 | 0,005 | **0.0007** | **0.009** | - | 0.318 |
| *Peptococcaceae* | 0,266 | 0,053 | 0,331 | 0,078 | 0,266 | 0,069 | 0,267 | 0,060 | 0,132 | 0,039 | | 0,211 | 0,032 | 0,504 | 0,103 | 0,126 | 0,078 | **0.006** | **0.034** | - | 0.252 |
| *Peptoniphilaceae* | 0,032 | 0,017 | 0,132 | 0,120 | 0,056 | 0,052 | 0,022 | 0,022 | 0,000 | 0,000 | | 0,007 | 0,007 | 0,057 | 0,032 | 0,000 | 0,000 | 0.175 | 0.444 | - | 0.130 |
| *Peptostreptococcaceae* | 0,422 | 0,132 | 0,099 | 0,044 | 0,320 | 0,157 | 0,965 | 0,338 | 0,251 | 0,141 | | 0,287 | 0,064 | 0,267 | 0,094 | 0,143 | 0,053 | **0.031** | 0.155 | - | 0.195 |
| *Porphyromonadaceae* | 13,144 | 1,812 | 10,244 | 1,325 | 10,246 | 0,770 | 13,984 | 2,093 | 11,659 | 1,719 | | 13,067 | 1,164 | 13,405 | 1,068 | 9,057 | 0,805 | - | **Phe x THC x CBD 0.006** | **Phe x THC x CBD**  **0.003** | 0.138 |
| *Prevotellaceae* | 10,277 | 1,778 | 12,190 | 1,033 | 9,712 | 1,127 | 8,914 | 1,910 | 14,629 | 2,633 | | 13,780 | 1,793 | 11,332 | 0,963 | 13,324 | 2,258 | - | **CBD 0.044** | **CBD 0.022** | 0.115 |
| *Rikenellaceae* | 1,272 | 0,249 | 1,290 | 0,234 | 1,473 | 0,404 | 2,451 | 0,475 | 1,806 | 0,447 | | 1,566 | 0,426 | 1,052 | 0,164 | 1,451 | 0,311 | 0.539 | 0.687 | - | 0.076 |
| *Ruminococcaceae* | 9,876 | 1,805 | 13,580 | 2,814 | 10,797 | 2,228 | 7,962 | 1,707 | 5,837 | 0,949 | | 7,684 | 0,985 | 11,123 | 1,048 | 11,245 | 1,046 | **0.032** | 0.155 | - | 0.195 |
| *Sphingobacteriaceae* | 0,728 | 0,259 | 0,209 | 0,082 | 0,170 | 0,038 | 0,218 | 0,067 | 0,162 | 0,064 | | 0,227 | 0,066 | 0,245 | 0,073 | 0,165 | 0,042 | 0.459 | 0.750 | - | 0.085 |
| *Streptococcaceae* | 0,089 | 0,043 | 0,051 | 0,023 | 0,032 | 0,019 | 0,023 | 0,012 | 0,056 | 0,036 | | 0,038 | 0,020 | 0,084 | 0,031 | 0,000 | 0,000 | 0.066 | 0.226 | - | 0.168 |
| *Sutterellaceae* | 0,394 | 0,081 | 0,220 | 0,052 | 0,293 | 0,089 | 0,255 | 0,074 | 0,186 | 0,114 | | 0,335 | 0,134 | 0,318 | 0,051 | 0,152 | 0,052 | 0.111 | 0.315 | - | 0.148 |
| *Thermoactinomycetaceae* | 0,146 | 0,102 | 0,256 | 0,096 | 0,092 | 0,043 | 0,007 | 0,007 | 0,037 | 0,030 | | 0,045 | 0,027 | 0,238 | 0,115 | 0,217 | 0,148 | 0.229 | 0.509 | - | 0.118 |
| *Thermoanaerobacteraceae* | 0,148 | 0,045 | 0,067 | 0,040 | 0,062 | 0,035 | 0,002 | 0,001 | 0,016 | 0,011 | | 0,018 | 0,014 | 0,043 | 0,035 | 0,000 | 0,000 | **0.0003** | **0.008** | - | 0.341 |
| *unclassified Clostridiales* | 0,279 | 0,103 | 0,201 | 0,080 | 0,286 | 0,091 | 0,323 | 0,089 | 0,181 | 0,081 | | 0,151 | 0,072 | 0,299 | 0,104 | 0,164 | 0,067 | 0.509 | 0.797 | - | 0.079 |
| *Veillonellaceae* | 0,281 | 0,078 | 0,373 | 0,147 | 0,241 | 0,055 | 0,750 | 0,390 | 0,281 | 0,063 | | 0,291 | 0,040 | 0,288 | 0,052 | 0,160 | 0,036 | 0.251 | 0.540 | - | 0.114 |
| *Verrucomicrobiaceae* | 0,072 | 0,043 | 0,000 | 0,000 | 0,000 | 0,000 | 0,069 | 0,038 | 0,000 | 0,000 | | 0,099 | 0,053 | 0,032 | 0,032 | 0,000 | 0,000 | **0.006** | **0.028** | - | 0.250 |
