# Supplementary material for: THC and CBD differentially modulated gut microbiota in the Poly I:C rat model of schizophrenia
Source: Front Pharmacol. 2026 Aug 10;17:1858604. doi: 10.3389/fphar.2026.1858604 (PMC13500884; doi:10.3389/fphar.2026.1858604)
Supplement: Supplementary file 3 [file Table3.docx]

**Supplementary Table 3.** **Data from genus-species populations**. Table shows mean ± SEM per group. Statistical comparisons were performed using Kruskal-Wallis (KW) or 3-way ANOVA as appropriate.

FDR-adjusted q-values were calculated using the Benjamini-Hochberg procedure. *p < 0.05; **p < 0.01; ***p < 0.001. Effect size is expressed as η².

| Genus-species | Sal  VH-VH | | MIA  VH-VH | | Sal  THC-VH | | MIA  THC-VH | | Sal  VH-CBD | | MIA  VH-CBD | | Sal  THC-CBD | | MIA  THC-CBD | | KW  (p- value) | FDR  (q- value) | 3-way ANOVA  (p- value) | η2 |
| --- | --- | --- | --- | --- | --- | --- | --- | --- | --- | --- | --- | --- | --- | --- | --- | --- | --- | --- | --- | --- |
|  | **Mean** | **SEM** | **Mean** | **SEM** | **Mean** | **SEM** | **Mean** | **SEM** | **Mean** | **SEM** | **Mean** | **SEM** | **Mean** | **SEM** | **Mean** | **SEM** |  |  |  |  |
| *[Ruminococcus]* | 6,009 | 1,239 | 7,530 | 0,997 | 7,883 | 0,787 | 8,520 | 1,377 | 8,960 | 1,150 | 7,333 | 0,713 | 8,396 | 1,106 | 11,347 | 1,116 | **0.049** | 0.121 | - | 0.179 |
| *[Ruminococcus] gnavus* | 1,149 | 0,248 | 1,980 | 0,271 | 2,289 | 0,581 | 2,241 | 0,516 | 3,025 | 0,497 | 1,819 | 0,354 | 2,719 | 0,601 | 2,382 | 0,529 | **-** | 0.079 | CBD 0.084  Phe x CBD 0.079 | 0.127 |
| *Akkermansia* | 0,072 | 0,043 | 0,000 | 0,000 | 0,000 | 0,000 | 0,069 | 0,038 | 0,000 | 0,000 | 0,099 | 0,053 | 0,032 | 0,032 | 0,000 | 0,000 | **0.006** | **0.022** | **-** | 0.250 |
| *Akkermansia muciniphila* | 0,072 | 0,043 | 0,000 | 0,000 | 0,000 | 0,000 | 0,068 | 0,037 | 0,000 | 0,000 | 0,099 | 0,053 | 0,032 | 0,032 | 0,000 | 0,000 | **0.006** | **0.022** | - | 0.250 |
| *Alistipes* | 0,785 | 0,208 | 1,097 | 0,212 | 1,102 | 0,359 | 1,890 | 0,336 | 1,426 | 0,357 | 1,044 | 0,376 | 0,845 | 0,149 | 1,129 | 0,275 | 0.287 | 0.375 | **-** | 0.108 |
| *Anaerostipes* | 0,198 | 0,095 | 0,100 | 0,090 | 0,285 | 0,161 | 0,081 | 0,025 | 0,095 | 0,070 | 0,058 | 0,029 | 0,000 | 0,000 | 0,074 | 0,043 | 0.086 | 0.176 | - | 0.158 |
| *Bacteroides* | 0,279 | 0,074 | 0,205 | 0,104 | 0,438 | 0,126 | 0,458 | 0,193 | 0,231 | 0,078 | 0,223 | 0,048 | 0,058 | 0,040 | 0,158 | 0,083 | 0.060 | 0.134 | **-** | 0.171 |
| *Bacteroides uniformis* | 0,180 | 0,054 | 0,075 | 0,035 | 0,089 | 0,032 | 0,189 | 0,144 | 0,091 | 0,036 | 0,081 | 0,033 | 0,017 | 0,007 | 0,014 | 0,009 | 0.158 | 0.240 | - | 0.134 |
| *Bilophila* | 1,175 | 0,296 | 1,291 | 0,718 | 0,806 | 0,418 | 0,069 | 0,046 | 0,027 | 0,012 | 0,034 | 0,022 | 0,124 | 0,043 | 0,141 | 0,072 | **0.0003** | **0.005** | **-** | 0.343 |
| *Bilophila wadsworthia* | 1,175 | 0,296 | 1,289 | 0,717 | 0,806 | 0,418 | 0,068 | 0,046 | 0,027 | 0,012 | 0,034 | 0,022 | 0,124 | 0,043 | 0,141 | 0,072 | **0.0003** | **0.004** | - | 0.344 |
| *Blautia* | 0,093 | 0,072 | 0,014 | 0,014 | 0,006 | 0,006 | 0,012 | 0,011 | 0,007 | 0,007 | 0,015 | 0,010 | 0,005 | 0,005 | 0,010 | 0,010 | 0.895 | 0.895 | **-** | 0.037 |
| *Butyrivibrio* | 0,002 | 0,002 | 0,012 | 0,012 | 0,105 | 0,091 | 0,121 | 0,113 | 0,000 | 0,000 | 0,000 | 0,000 | 0,000 | 0,000 | 0,000 | 0,000 | 0.329 | 0.418 | - | 0.102 |
| *Butyrivibrio crossotus* | 0,002 | 0,002 | 0,000 | 0,000 | 0,100 | 0,087 | 0,106 | 0,098 | 0,000 | 0,000 | 0,000 | 0,000 | 0,000 | 0,000 | 0,000 | 0,000 | 0.185 | 0.264 | - | 0.127 |
| *Clostridium* | 6,661 | 0,785 | 5,332 | 0,823 | 7,760 | 0,825 | 5,928 | 0,833 | 4,347 | 0,739 | 4,888 | 0,751 | 4,854 | 0,467 | 4,686 | 0,971 | **-** | **CBD 0.002** | **CBD 0.0007**  Phe x CBD 0.067 | 0.175 |
| *Clostridium hiranonis* | 0,088 | 0,025 | 0,000 | 0,000 | 0,029 | 0,016 | 0,092 | 0,028 | 0,032 | 0,022 | 0,020 | 0,013 | 0,030 | 0,030 | 0,012 | 0,009 | **0.003** | **0.014** | - | 0.278 |
| *Coprococcus* | 0,123 | 0,055 | 0,251 | 0,125 | 0,365 | 0,221 | 0,249 | 0,203 | 0,834 | 0,335 | 0,193 | 0,125 | 0,447 | 0,151 | 0,203 | 0,148 | 0.273 | 0.367 | **-** | 0.110 |
| *Desulfovibrio* | 0,013 | 0,009 | 0,065 | 0,056 | 0,272 | 0,110 | 0,294 | 0,160 | 0,150 | 0,140 | 0,074 | 0,032 | 0,026 | 0,018 | 0,131 | 0,083 | 0.092 | 0.180 | - | 0.155 |
| *Desulfovibrio C21_c20* | 0,013 | 0,009 | 0,065 | 0,056 | 0,272 | 0,110 | 0,292 | 0,158 | 0,150 | 0,140 | 0,074 | 0,032 | 0,026 | 0,018 | 0,131 | 0,083 | 0.092 | 0.173 | - | 0.155 |
| *Eubacterium* | 0,056 | 0,026 | 0,106 | 0,041 | 0,273 | 0,129 | 0,187 | 0,065 | 0,061 | 0,045 | 0,119 | 0,052 | 0,213 | 0,084 | 0,074 | 0,043 | 0.495 | 0.541 | **-** | 0.081 |
| *Eubacterium hadrum* | 0,014 | 0,011 | 0,071 | 0,042 | 0,051 | 0,030 | 0,087 | 0,046 | 0,018 | 0,018 | 0,049 | 0,038 | 0,161 | 0,078 | 0,014 | 0,010 | 0.719 | 0.751 | - | 0.057 |
| *Eubacterium xylanophilum* | 0,036 | 0,024 | 0,018 | 0,018 | 0,155 | 0,107 | 0,048 | 0,040 | 0,019 | 0,019 | 0,000 | 0,000 | 0,029 | 0,020 | 0,000 | 0,000 | 0.446 | 0.511 | - | 0.087 |
| *Faecalibacterium* | 4,221 | 0,974 | 5,127 | 1,053 | 4,831 | 1,246 | 1,928 | 0,807 | 1,859 | 0,338 | 2,027 | 0,327 | 4,214 | 0,529 | 3,590 | 0,753 | **0.013** | **0.038** | **-** | 0.226 |
| *Faecalibacterium prausnitzii* | 0,349 | 0,171 | 0,157 | 0,030 | 0,185 | 0,036 | 0,116 | 0,053 | 0,054 | 0,029 | 0,101 | 0,041 | 0,135 | 0,046 | 0,124 | 0,040 | 0.058 | 0.136 | - | 0.173 |
| *Limosilactobacillus* | 4,191 | 0,394 | 5,018 | 1,238 | 5,115 | 1,588 | 7,766 | 1,794 | 16,125 | 8,970 | 7,355 | 0,994 | 7,360 | 2,310 | 5,939 | 1,951 | 0.242 | 0.335 | - | 0.116 |
| *Limosilactobacillus animalis* | 0,013 | 0,009 | 0,031 | 0,016 | 0,022 | 0,017 | 0,107 | 0,077 | 0,084 | 0,060 | 0,037 | 0,033 | 0,019 | 0,014 | 0,179 | 0,148 | 0.813 | 0.831 | **-** | 0.047 |
| *Limosilactobacillus hominis* | 0,000 | 0,000 | 0,009 | 0,009 | 0,011 | 0,011 | 0,035 | 0,022 | 0,462 | 0,384 | 0,029 | 0,022 | 0,004 | 0,004 | 0,024 | 0,012 | 0.469 | 0.525 | - | 0.084 |
| *Limosilactobacillus intestinalis* | 0,619 | 0,209 | 1,091 | 0,357 | 0,925 | 0,240 | 0,958 | 0,171 | 2,167 | 0,719 | 2,933 | 0,532 | 1,814 | 0,708 | 1,247 | 0,349 | **0.011** | **0.037** | **-** | 0.232 |
| *Limosilactobacillus johnsonii* | 0,536 | 0,161 | 0,149 | 0,059 | 0,306 | 0,128 | 0,572 | 0,227 | 0,560 | 0,170 | 0,600 | 0,239 | 0,468 | 0,200 | 0,158 | 0,056 | 0.163 | 0.239 | - | 0.133 |
| *Limosilactobacillus murinus* | 0,427 | 0,100 | 0,713 | 0,491 | 0,607 | 0,327 | 0,775 | 0,222 | 0,553 | 0,265 | 0,297 | 0,101 | 0,480 | 0,226 | 0,379 | 0,214 | 0.496 | 0.530 | - | 0.081 |
| *Limosilactobacillus reuteri* | 0,065 | 0,027 | 0,082 | 0,046 | 0,043 | 0,029 | 0,090 | 0,024 | 2,030 | 1,935 | 0,067 | 0,023 | 0,056 | 0,020 | 0,024 | 0,018 | 0.342 | 0.412 | **-** | 0.100 |
| *Limosilactobacillus vaginalis* | 0,277 | 0,054 | 0,193 | 0,051 | 0,253 | 0,112 | 0,727 | 0,165 | 1,936 | 1,143 | 0,606 | 0,047 | 0,504 | 0,152 | 0,328 | 0,176 | **0.0005** | **0.005** | - | 0.327 |
| *Mucispirillum* | 0,217 | 0,142 | 0,000 | 0,000 | 0,109 | 0,068 | 0,048 | 0,034 | 0,000 | 0,000 | 0,000 | 0,000 | 0,000 | 0,000 | 0,000 | 0,000 | **0.0008** | **0.006** | **-** | 0.314 |
| *Mucispirillum schaedleri* | 0,217 | 0,142 | 0,000 | 0,000 | 0,109 | 0,068 | 0,048 | 0,034 | 0,000 | 0,000 | 0,000 | 0,000 | 0,000 | 0,000 | 0,000 | 0,000 | **0.0008** | **0.005** | - | 0.314 |
| *Oribacterium* | 0,040 | 0,028 | 0,083 | 0,042 | 0,038 | 0,018 | 0,040 | 0,025 | 0,028 | 0,021 | 0,224 | 0,077 | 0,049 | 0,022 | 0,044 | 0,027 | 0.147 | 0.230 | - | 0.137 |
| *Oribacterium sinus* | 0,006 | 0,006 | 0,017 | 0,017 | 0,038 | 0,018 | 0,036 | 0,023 | 0,021 | 0,021 | 0,198 | 0,083 | 0,005 | 0,005 | 0,020 | 0,015 | 0.132 | 0.214 | **-** | 0.141 |
| *Oscillibacter* | 6,009 | 1,239 | 0,066 | 0,023 | 0,066 | 0,014 | 0,481 | 0,227 | 0,552 | 0,116 | 0,494 | 0,077 | 0,171 | 0,054 | 0,592 | 0,091 | **<0.0001** | **0.001** | - | 0.593 |
| *Oscillibacter valericigenes* | 0,009 | 0,006 | 0,019 | 0,010 | 0,006 | 0,003 | 0,105 | 0,029 | 0,215 | 0,074 | 0,175 | 0,032 | 0,051 | 0,024 | 0,246 | 0,066 | **<0.0001** | **0.002** | **-** | 0.595 |
| *Parabacteroides* | 0,206 | 0,075 | 0,168 | 0,115 | 0,000 | 0,000 | 0,335 | 0,170 | 0,008 | 0,006 | 0,022 | 0,019 | 0,052 | 0,024 | 0,007 | 0,007 | **0.003** | **0.016** | - | 0.271 |
| *Parabacteroides distasonis* | 0,175 | 0,069 | 0,159 | 0,106 | 0,000 | 0,000 | 0,231 | 0,101 | 0,005 | 0,005 | 0,022 | 0,019 | 0,028 | 0,015 | 0,007 | 0,007 | **0.002** | **0.012** | - | 0.291 |
| *Parasutterella* | 0,394 | 0,081 | 0,220 | 0,052 | 0,293 | 0,089 | 0,255 | 0,074 | 0,186 | 0,114 | 0,335 | 0,134 | 0,318 | 0,051 | 0,152 | 0,052 | 0.111 | 0.186 | **-** | 0.148 |
| *Parasutterella excrementihominis* | 0,394 | 0,081 | 0,220 | 0,052 | 0,293 | 0,089 | 0,253 | 0,072 | 0,186 | 0,114 | 0,335 | 0,134 | 0,318 | 0,051 | 0,152 | 0,052 | 0.111 | 0.193 | - | 0.148 |
| *Prevotella* | 2,357 | 0,492 | 3,676 | 0,632 | 1,947 | 0,350 | 2,951 | 0,655 | 3,990 | 1,011 | 3,465 | 0,505 | 3,099 | 0,505 | 2,281 | 0,461 | **-** | 0.078 | THC 0.052  Phe x CBD 0.052 | 0.123 |
| *Roseburia* | 0,268 | 0,147 | 0,185 | 0,165 | 0,214 | 0,080 | 0,513 | 0,363 | 0,025 | 0,013 | 0,054 | 0,034 | 0,034 | 0,018 | 0,000 | 0,000 | **0.003** | **0.013** | **-** | 0.274 |
| *Roseburia faecis* | 0,094 | 0,059 | 0,107 | 0,107 | 0,090 | 0,048 | 0,301 | 0,206 | 0,000 | 0,000 | 0,032 | 0,027 | 0,025 | 0,013 | 0,000 | 0,000 | **0.005** | **0.020** | - | 0.258 |
| *Ruminiclostridium* | 0,050 | 0,016 | 0,043 | 0,013 | 0,086 | 0,020 | 0,138 | 0,031 | 0,072 | 0,021 | 0,115 | 0,036 | 0,050 | 0,025 | 0,021 | 0,011 | **0.032** | 0.084 | **-** | 0.194 |
| *Ruminococcus* | 0,635 | 0,116 | 0,838 | 0,259 | 0,891 | 0,133 | 1,595 | 0,237 | 0,577 | 0,197 | 1,008 | 0,132 | 1,094 | 0,195 | 0,720 | 0,173 | **0.012** | **0.038** | **-** | 0.229 |
| *Ruminococcus bromii* | 0,062 | 0,027 | 0,100 | 0,036 | 0,043 | 0,019 | 0,111 | 0,045 | 0,028 | 0,013 | 0,062 | 0,018 | 0,044 | 0,018 | 0,039 | 0,029 | 0.408 | 0.479 | - | 0.091 |
| *Ruminococcus flavefaciens* | 0,164 | 0,051 | 0,253 | 0,100 | 0,192 | 0,069 | 0,387 | 0,101 | 0,397 | 0,153 | 0,481 | 0,104 | 0,606 | 0,122 | 0,422 | 0,133 | 0.099 | 0.179 | - | 0.153 |
| *Ruminococcus gnavus* | 0,147 | 0,049 | 0,075 | 0,041 | 0,285 | 0,080 | 0,505 | 0,260 | 0,086 | 0,039 | 0,146 | 0,024 | 0,069 | 0,034 | 0,086 | 0,030 | **0.030** | 0.083 | **-** | 0.196 |
| *Turicibacter* | 0,235 | 0,078 | 0,040 | 0,026 | 0,127 | 0,077 | 0,281 | 0,128 | 0,065 | 0,059 | 0,163 | 0,073 | 0,219 | 0,168 | 0,284 | 0,170 | 0.336 | 0.416 | - | 0.101 |
