# Supplementary material for: THC and CBD differentially modulated gut microbiota in the Poly I:C rat model of schizophrenia
Source: Front Pharmacol. 2026 Aug 10;17:1858604. doi: 10.3389/fphar.2026.1858604 (PMC13500884; doi:10.3389/fphar.2026.1858604)
Supplement: Supplementary file 4 [file Table4.docx]

**Supplementary Table 4.** **Top Spearman correlations between microbiota phyla and IOS markers (iNOS, NQO1, and HO-1) across experimental groups (p < 0.1).** Correlations are shown only for associations with p<0.1. Positive values indicate that both variables changed in the same direction, whereas negative values indicate inverse associations.

| Group | Bacteria | IOS | Spearman | p-value |
| --- | --- | --- | --- | --- |
| Saline VH-VH | Bacillota | HO1 | -0.678571 | 0.09375 |
| MIA VH-VH | Mycoplasmatota | iNOS | 0.678571 | 0.09375 |
|  | Mycoplasmatota | HO1 | -0.785714 | 0.036238 |
| Saline THC-VH | Mycoplasmatota | iNOS | 0.809524 | 0.014903 |
|  | Unclassified | iNOS | 0.761019 | 0.028298 |
|  | Actinomycetota | NQO1 | -0.627376 | 0.095891 |
|  | Unclassified | NQO1 | -0.684917 | 0.060886 |
|  | Deferribacterota | iNOS | -0.79104 | 0.019385 |
|  | Pseudomonadota | iNOS | -0.880952 | 0.00385 |
| MIA THC-VH | Nitrospinae | iNOS | 0.650613 | 0.080638 |
|  | Actinomycetota | iNOS | 0.634335 | 0.091163 |
|  | Mycoplasmatota | NQO1 | -0.666667 | 0.070988 |
|  | Unclassified | HO1 | -0.797922 | 0.01763 |
|  | Mycoplasmatota | HO1 | -0.857143 | 0.00653 |
|  | Unclassified | NQO1 | -0.896127 | 0.002588 |
| Saline VH-CBD | Pseudomonadota | NQO1 | 0.809524 | 0.014903 |
|  | Nitrospinota | HO1 | -0.627376 | 0.095891 |
| MIA VH-CBD | Nitrospinota | iNOS | 0.867533 | 0.005249 |
|  | Bacteroidetes | iNOS | 0.714286 | 0.046528 |
|  | Mycoplasmatota | HO1 | 0.642857 | 0.085559 |
|  | Nitrospinota | HO1 | -0.65065 | 0.080615 |
|  | Bacillota | iNOS | -0.690476 | 0.05799 |
|  | Mycoplasmatota | iNOS | -0.785714 | 0.020815 |
|  | Verrucomicrobiota | iNOS | -0.79104 | 0.019385 |
| Saline THC-CBD | -- | -- | -- | -- |
