# Supplementary material for: THC and CBD differentially modulated gut microbiota in the Poly I:C rat model of schizophrenia
Source: Front Pharmacol. 2026 Aug 10;17:1858604. doi: 10.3389/fphar.2026.1858604 (PMC13500884; doi:10.3389/fphar.2026.1858604)
Supplement: Supplementary file 5 [file Table5.docx]

**Supplementary Table 5. Top Spearman correlation values between gut microbiota phyla and ventricular volume across experimental groups.** Correlations are shown only for associations with p<0.1. Positive values indicate that both variables changed in the same direction, whereas negative values indicate inverse associations.

| Group | Bacteria | Spearman | p-value |
| --- | --- | --- | --- |
| MIA VH-VH | Unclassified | 0.631558 | 0.050161 |
|  | Actinomycetota | 0.566715 | 0.087601 |
| Saline THC-VH | Unclassified | 0.757896 | 0.011089 |
|  | Mycoplasmatota | 0.551515 | 0.098401 |
|  | Deferribacterota | -0.58037 | 0.078574 |
| MIA THC-VH | Bacillota | 0.575758 | 0.081553 |
|  | Bacteroidota | -0.684848 | 0.028883 |
| Saline VH-CBD | Mycoplasmatota | 0.638301 | 0.047024 |
|  | Bacteroidota | 0.563636 | 0.089724 |
|  | Bacillota | -0.551515 | 0.098401 |
|  | Actinomycetota | -0.588322 | 0.073606 |
| MIA VH-CBD | Mycoplasmatota | -0.551515 | 0.098401 |
| MIA THC-CBD | Mycoplasmatota | -0.729487 | 0.016647 |
